# Meeting report of the eight annual Tri-Service Microbiome Consortium Symposium

**DOI:** 10.1186/s12919-025-00355-z

**Published:** 2025-12-23

**Authors:** Melissa R. Kardish, Richard T. Agans, Robyn A. Barbato, Stacey J. Doherty, Michael S. Goodson, J. Philip Karl, Robert Kokoska, Zachary S. Liechty, Neal D. McNeal, Kenneth Racicot, J. Jordan Steel, Charlie Sweet, Sara M. Tuck, Dagmar H. Leary

**Affiliations:** 1 United States Naval Research Laboratory, 4555 Overlook Ave SW, , Washington, D.C. 20375 USA; 2 711, Human Performance Wing, Air Force Research Laboratory, Wright‐ Patterson AFB, Dayton, OH USA; 3 US Army Engineer Research and Development Center, Cold Regions Research and Engineering, Hanover, NH USA; 4 United States Army Research Institute of Environmental Medicine, Natick, MA USA; 5 DEVCOM-ARL-Army Research Office, Research Triangle Park, Durham, NC USA; 6 Health and Performance Technologies Division, Blue Halo, Inc., Dayton, OH USA; 7 Naval Submarine Medical Research Laboratory, Groton, CO USA; 8 Soldier Effectiveness Directorate, United States Army Combat Capabilities Development Command Soldier Center, Natick, MA USA; 9 United States Air Force Academy, Colorado Springs, CO USA; 10 Unites States Naval Academy, Annapolis, MD USA; 11 NMRC, Silver Spring, MD USA

## Abstract

The Tri-Service Microbiome Consortium (TSMC) was created to foster and enhance cooperation, collaboration, and communication of microbiome research among Department of Defense (DoD) researchers and their collaborators. The 8th Annual TSMC Symposium was held in Colorado Springs, CO on 25–26 September 2024 and featured oral and poster presentations and discussions centered on microbiome-related topics within four broad thematic areas: 1) Surveillance; 2) Health and Performance; 3) Enablers; and 4) Remediation. Collectively this meeting promoted sharing of methods, experiments, and findings across DoD affiliated microbiome research and promoted future and ongoing collaborations. This report summarizes the proceedings of the 8th Annual TSMC Symposium.

**Supplementary Information:**

The online version contains supplementary material available at 10.1186/s12919-025-00355-z.

## Introduction

The Tri-Service Microbiome Consortium (TSMC) was established in 2016 to enhance collaboration, coordination, and communication of microbiome research among Department of Defense (DoD) organizations and to facilitate resource, material, and information sharing among consortium members extending to industry, academia, and other governmental organizations [1–8]. The 8th annual TSMC symposium was a hybrid meeting held in Colorado Springs, CO, from 25–26 September 2024. Of the 170 registered attendees, 79% were from DoD institutions (35 institutions represented, including three military academies), 10% from non-DoD government (nine institutions), 5% from industry partners (six institutions), 4% from academia (seven institutions), and 2% from foreign governments (three institutions). The two-day meeting featured 20 research talks, three special sessions on funding, data resources, and customer (i.e., military) insights and experiences, and 24 posters. Research talks were focused in four sessions: 1) Surveillance; 2) Health and Performance; 3) Enablers; and 4) Remediation. Speakers for research talks were largely from DoD institutions (70%) and 60% were early career scientists. Two-thirds of the posters came from DoD presenters, 5 of which were presented by cadets and midshipmnet. The remaining posters were from academic, industry and non-DoD government partners. In this report, we detail the research presented at the 8th annual TSMC symposium to facilitate communication of current and ongoing research efforts in the DoD community and enable ongoing and future collaborations.

### Surveillance

The Surveillance session discussed ongoing research efforts broadly related to the surveillance of pathogens, pollutants, and microbial communities in different environments. Specific topics included, validating pathogen diagnostic panels, the power of wastewater-based epidemiology to provide surveillance data on whole populations, the impact of chemical pollutants on environmental microbial communities, and the impact of active layer soil and permafrost freeze–thaw and mixing dynamics on microbial metabolism and activity.

Respiratory and gastrointestinal infectious diseases have significant impacts on the overall health and readiness of active-duty military personnel, resulting in over 95,000 lost duty days annually. The Defense Center for Public Health-Dayton Epidemiology Laboratory serves as the DoD’s reference lab, processing more than 12,000 clinical specimens each year. However, in 67% of samples a causative agent cannot be identified by the certified panels being used for screening samples. A limitation of traditional screening using certified panels is a reliance on the BioFire PCR diagnostic instrument that performs directed PCR for specific disease targets [9]. This direct PCR uses gene-specific primers to amplify a gene target, which inherently is not able to detect mutant strains that contain genetic mutations in the primer binding sites. USAF School or Aerospace Medicine’s Applied Genomics and Technology Laboratory (USAFSAM) is employing metagenomic shotgun sequencing to profile microbial communities in respiratory and fecal samples. The research aims to correlate microbial profiles with pathogen detection and demographic data to glean insights on health status and geographic factors corresponding to different microbial profiles. Additionally, the metagenomic shotgun data will be able to detect the whole community of microbes and may provide a comprehensive picture of the infection, rather than simple targeted PCR analysis. Ultimately the research aims to identify complex microbial interactions impacting military health.

Emerging viruses are difficult to trace, warranting environmental surveillance to track pathogens. Because microbial particles are shed in human waste, wastewater can be used to detect emerging pathogens such as SARS-CoV2 [10]. U.S. military members face heightened risks from transmissible pathogens, especially in congregate settings, which can lead to significant operational disruptions [11]. Research on integrating wastewater surveillance with clinical data to enhance monitoring of respiratory pathogens, a method that proved effective during the COVID-19 pandemic, was presented. Defense Centers for Public Health Aberdeen and USAFSAM have successfully collected wastewater from the US Naval Academy and screened for pathogens. The presented research discussed efforts to collaboratively expand wastewater screening to profile respiratory viruses and bacterial pathogens in wastewater around DOD military bases and establish a predictive framework by correlating wastewater data with clinical case trends. The overall aim is to enhance the understanding of respiratory pathogen dynamics and informing public health strategies in military settings.

Environmental pollutants have detrimental effects on biodiversity, particularly among microbial communities [12]. To better assess that relationship, sediment and water samples were collected from eight sites in the Great Lakes region and were analyzed using shotgun metagenomic sequencing and chemical assessments for over 200 pollutants. Results showed that the concentrations of pollutants in sediment were significantly greater than in water samples. Different chemicals were detected in water versus sediment samples: 58.2% of chemical pollutants were found exclusively in the water, 15.8% of chemicals were unique to sediment, and 26% of chemicals were found in both sediment and water samples. Using total shotgun sequences to compare microbial communities in the water and the sediment revealed a decrease in microbiota density of protozoan, metazoan, and fungal species and an increase in certain bacterial and archaeal populations in response to higher chemical pollutant concentrations. Overall, the results suggest that microbial diversity is significantly impacted by chemical pollutant concentration and warrants consideration as a proxy for assessing chemical toxicity levels in the environment.

Molecular techniques and carbon use assays are being used to survey microbial communities in Arctic soils. These Arctic soils are rapidly degrading due to warming posing challenges for military operations in cold regions and also has significant impacts to microbial communities in seasonally thawed active layer and permafrost [13, 14]. The final study in this session examined how mixing active layer and permafrost soils affects microbial community structure and carbon utilization. It was hypothesized that adding active layer soil would enhance carbon utilization. Findings showed that higher respiration rates and a greater diversity of carbon substrate utilization occurred in soils with more active layer content. The mixed communities resembled those of active layer soils rather than those from the transition zone, highlighting the importance of understanding soil mixing effects for predicting carbon-climate interactions and microbial community impacts [15].

### Health and performance

The symposium had two Health and Performance sessions. The first session covered the interactions of the gut microbiome with various stressors, including acute stress in relation to Post-Traumatic Stress Disorder (PTSD) development, acute mountain sickness, and traumatic brain injuries. Overviews of broad efforts by the United States Department of Veterans Affairs and the Defense Science and Technology Laboratory (DSTL) in the United Kingdom’s Ministry of Defense were also discussed.

Military trauma involves repeated acute stress exposures causing a catecholamine surge that activates a biological cascade which can contribute to the development of PTSD [16, 17]. Treatment of these symptoms while deployed is challenging due to austere conditions and resource and personnel limitations. The microbiome may be involved with the downstream molecular cascade and understanding the interplay of the microbiome with stress responses presents an opportunity to interrupt the cascade and provide resilience to stressors. Researchers sought to investigate the role of the gut microbiome during stress exposure using a Sprague–Dawley rat model. Two cohorts of male and female mice were exposed to a series of acute stressors, including: predator exposure, foot shock, and underwater stress. After 24 h, anxiety behavior was tested using an elevated plus maze, open field, and acoustic startle response. The behavioral tests revealed that both males and females had anxiety-relevant behavioral stress responses in the elevated plus maze; however, males but not females displayed elevated anxiety-like behavioral response in the open field test when compared to non-stressed mice. The degree of altered behavior was greater in males compared to females among the first cohort. The first cohort had a significant reduction in microbiome alpha diversity in stress conditions. However, neither of these results were replicated in the second cohort. Notably, Cohort 1 had multiple Gammaproteobacteria that responded to stress in a sex-dependent fashion whereas Cohort 2 had a greater abundance of Gammaproteobacteria in the initial microbiome. These differences, might explain the differing responses of the cohorts to stress treatment and suggest the composition of pre-exposed gut microbiome could influence community responses to military relevant stressors.

Relations between the gut microbiome and physiological stress were further explored in an investigation of the associations between the gut microbiome and acute mountain sickness (AMS). The partial pressure of oxygen in the atmosphere decreases as altitude increases. This results in insufficient oxygen being delivered to bodily tissues and a cascade of physiologic responses can contribute to the development of altitude illnesses including AMS. AMS symptoms can range from mild to severe, and include headaches, dizziness, gastrointestinal distress, and fatigue [18, 19]. There is high inter-individual variability in AMS severity, and the researchers hypothesized that inter-individual variability within gut microbiomes could play a role. Seventy-five participants donated fecal samples before a four-day, high-altitude sojourn. 57% of respondents experienced no AMS symptoms whereas 19% experienced moderate or severe symptoms. Participants who had moderate or severe symptoms had a lower microbiome alpha diversity and a significantly different beta diversity compared to participants who were not sick, with more severe sickness associated with an increased abundance of certain *Bacteroides* species. AMS severity was also associated with bacterial gene pathways related to lipopolysaccharide biosynthesis and amino acid and energy metabolism. Findings suggested that the gut microbiome may be a factor contributing to the development of AMS during high altitude sojourn.

The session then transitioned to broader efforts to study the microbiome in relation to health and performance in military personnel. A representative from the United Kingdom’s Defence Science and Technology Laboratory (DSTL) outlined the DSTL’s microbiome portfolio and project interfaces, which included Disease Non-Battle Injury, Optimizing Human Performance, and Human Augmentation. One study highlighted under the Disease Non-Battle Injury line of effort explored the differences in microbiomes of Gurkha (soldiers of Nepalese descent) and non-Gurkha soldiers [20]. Gurkha’s have a lower occurrence of Travelers’ Diarrhea compared to soldiers born in the UK [21], and understanding differences in their gut microbiomes could provide insight into resistance to Travelers’ Diarrhea. This study found the Gurkha population displayed a lower microbiome alpha diversity and increased abundance of *Haemophilus parainfluenzae*. Current and future projects under Optimizing Human Performance were outlined including a study examining changes to the gut microbiome throughout an Officer training course and investigating the effects of commercial pre- and probiotics on healthy personnel. Human Augmentations projects also included methods for rapid detection of shifts in microbiome composition and characterizing the respiratory microbiome of UK Ministry of Defense personnel.

The final broad effort discussed during the first Health and Performance session was that of the Military and Veteran Microbiome Consortium for Research and Education from the U.S. Department of Veterans Affairs. This is an ongoing study beginning in 2016, collecting skin, oral and gut microbiomes, clinical interviews, and mental health surveys from Veterans every six months. Over 6000 samples have been collected and 16S rRNA amplicon sequencing has been completed from over 4500 personnel. Numerous manuscripts have been published using the dataset since the project began on topics including probiotics, mild traumatic brain injuries (TBI) and PTSD, food deserts, and homelessness [22–24]. Much of this research focused on TBIs and mental health. Surveys found that Veterans with a history of TBI were more likely to experience mental health issues over time. Investigating the microbiomes of participants who did or did not experience TBIs revealed no differences in either microbiome alpha or beta diversity between the groups. However, in a follow up study, participants without a diagnosed psychological disorder yielded a greater ratio of *Faecalibacterium* to *Bacteroides* compared to participants who either had psychological disorders in the past or would go on to receive a diagnosis. A follow-up mechanistic study inducing TBIs in mice found that male subjects had greater decreases in alpha diversity and a larger beta diversity shift post TBI compared to female subjects. These studies can help further understand the risks for Veterans who experienced TBIs and help in the development of mitigation strategies.

The second Health and Performance session drew examples from swine wound models, melanin in the intestine, shigellosis, and alcohol consumption to highlight the intricate interplay between microbiomes and various health factors, presenting avenues for future research into innovative treatments and understanding the underlying mechanisms of microbial dynamics in health and disease.

Microbiome dynamics in the context of swine burn wounds was discussed by exploring how various topical agents aid in healing. Topical healing agents include antimicrobial ointments, such as silver-containing agents and bismuth-impregnated petroleum gauze, aimed at reducing inflammation and promoting recovery in partial thickness burns. These agents may also interact with the microbiome with effects on the healing process and microbiome recovery from burns [25, 26]. The cannabinoid treatment NEPE-14and its assessment for wound healing compared to standard treatments was the subject of the presented work. Results indicated that the cannabinoid treatment NEPE-14 plays a crucial role in reducing inflammation and mitigating burn progression. Notably, significant differences were observed between NEPE-14 and silver ion treatments across several weeks, suggesting that different therapies utilize distinct mechanisms of action, despite similar healing timelines. Significant temporal changes in microbiome diversity were also observed during NEPE-14 treatment, including an increase in Proteobacteria over time but the impact on wound healing is unclear.

Also discussed in this session was the protective role of melanized bacteria in intestine, particularly in the context of radiation exposure. Melanin, a component of fungal cell walls, provides substantial protection against radiation as evidenced by the discovery of black-pigmented microbes in high-radiation environments, such as Chernobyl and the International Space Station [27, 28]. In that work, *Exophiala lecanii-corni* was identified as a key fungal species, with melanin production linked to enhanced resistance to UV and gamma radiation, opening the possibility of developing melanized probiotics that could help protect against radiation-induced intestinal injuries, maintaining gut health and modulating microbiota. The discussed research aimed to apply synthetic biology to produce diverse and cost-effective melanins, potentially for military applications. Experiments demonstrated that melanins offered protective effects, and did not significantly alter the gut microbiome, suggesting the need for further exploration of potential military applications of melanized microbial strains.

Using a controlled human infection model of travelers’ diarrhea, researchers analyzed how *Shigella sonnei* colonization alters microbial dynamics of the gut microbiome [29]. Individuals experiencing shigellosis exhibit persistent changes in gut microbiota, with significant decreases in alpha diversity linked to the infection and subsequent antibiotic treatment. The presented study highlighted that those affected by shigellosis display differential abundance of specific microbial taxa and suggested that initial microbiome composition may influence susceptibility to infection. The long-term effects on gut health were underscored by persistent shifts in microbiome structure following infection, indicating the need for further research into recovery strategies.

The final presentation of the session addressed the effects of alcohol consumption on the gut microbiome, particularly among military personnel and veterans. This research underscored the complex relationship between alcohol use, mental health, and gut microbiome composition. Findings suggested that binge drinking is associated with a more significant impact on microbial diversity than moderate, consistent consumption. Further, the presented research identified individual variability in gut microbiome associations with alcohol consumption, influenced by factors such as homelessness and traumatic brain injury. These findings emphasize that lifestyle and socio-economic factors contribute significantly to microbiome health, complicating the understanding of alcohol’s effects.

### Enablers

The Enablers session focused on tools, techniques, and computational pipelines that increase the ability to work with and understand microbes. Collaborations among TSMC members from different institutions and DoD services have long been impeded by the speed of data exchange. This session was aimed to inform the membership about the available resources and directly introduce researchers involved in building these tools to the community.

The session opened with a presentation on how DoD high performance computing facilities are being employed to aid in designing high throughput tools to guide researchers in isolating, redesigning, and controlling microbes in austere and complex environments. The effort started as a response to increasing interest in finding the “unknown unknowns”, or microbes which researchers are not aware they do not know. In the first phase of this effort, researchers exposed material coupons to diverse environments and then profiled resulting microbes over time, in addition they characterized changes to the coupons themselves. In the second phase, researchers plan to utilize metagenomics approaches to characterize the resident microbiomes and assess any temporal changes to the consortium. Phase three will be phenotypic characterization following cultivation of microbial isolates obtained from coupons. Genome sequencing will be employed to enable mining for genes responsible for unique biochemical processes.

On the computational side, discussions centered on comparing informatic tools and pipelines, computing requirements, and how this integrates into handling of big data; all within DoD computational environments and constraints. Current progress included collection and banking of hundreds of isolates, with phenotypic characterization, and metagenomes representing multiple material types; with the computational resource improvement of genome analysis from 7 days down to 2 h.

An important component to complex microbiome characterization work is quality assessment and variant quantification, particularly when measuring 16S ribosomal RNA gene [30]. To address this, the Military and Veteran Microbiome Consortium for Research and Education compared the effects of collection, transport, and storage conditions on sequencing results and against commercial mock community standards. Over 400 participants contributed a total of over 3,000 oral and fecal samples over a period of 90 months. Results highlighted differences in members from Bacteroidetes, Proteobacteria, among others and between fecal samples collected using swabs versus the Omnigene kit, with preservative agents appearing to select for gram positive bacteria. Interestingly, sample transport time was associated with increased Proteobacteria and Gammaproteobacteria. DEBLUR [31] was used to correct these enrichments.

Advancements in computing technology and power have allowed concomitant advancements in the way DoD researchers apply computing to complex microbial community analyses, especially artificial intelligence and machine learning. To illustrate this, applications of neural networks to multiple different efforts to highlight AI/ML methods for data fusion of microbiome profiles and clinical assessments in deployed environments were highlighted. These methods integrate multimodal data including microbial profiles, metabolic classifications, metadata, and more, and then employ neural networks to characterize health status of military individuals [32]. One effort using this approach is determining microbial composition and function among submariners undergoing prolonged deployment. The network incorporated microbial profiles, cytokine blood panels, and abbreviated profile of mood states (POMS) responses. The network is first trained using 12,000 publicly available microbiome profiles which are characterized by disease status, disease type, and collection location. Further refinement was performed with submariner microbiome profiles, POMS responses, and blood panel data, which have all been pre-trained. Following iterations of training and fine-tuning, this network was used to predict and classify performance status utilizing microbiome data.

To round out the session, the focus shifted to physical tools that allow cultivation and examination of microbiomes. In particular, following the 2022 FDA rule enabling microphysiological systems to be used as pre-human models, the development of organoids and organ-on-chips has rapidly expanded. The ArtGut, a high-throughput artificial gut model designed to allow cultivation and co-culture of facultative and strict anaerobes. This device consists of a multi-well setup of human cell-types that can be co-cultured with microbiomes, allowing simultaneous replicate experiments to be performed. It has recently been applied to study gut microbiomes and organophosphate exposures, wherein complex microbiomes were incubated in the presence of organophosphates for upwards of 14 h. Interestingly, while few taxonomical changes were noted, there was a significant decrease in metabolites from tryptophan metabolic pathways and increased presence of isobutyric acid in cultures. This was followed by addition of L-tryptophan and vitamin E which resulted in a mitigation of the organophosphate responses to metabolites.

### Remediation

The fourth thematic session focused on potential remediation strategies involving native microbiomes indigenous to marine environments, soils, and aircraft surfaces.

Environmental microbiomes contribute to marine fouling on the surfaces of Naval vessels. The accumulation of biofilms on these surfaces presents adverse consequences including increased frictional drag and fuel consumption, the time and costs associated with cleaning [33], and a surface environment that becomes a vector for invasive species. Examining microbial interactions with coatings may provide a first step to controlling later macrofouling, however this remediative approach is complicated by the diversity of microbiomes based on geographic location, seasonal considerations, changes in microbiome composition depending on successional stage, and interactions between members of the fouling microbiome. As a first step, researchers set out to perform a systematic study to characterize microbial communities on 12 different surface coatings (some including releasable biocide) at six different global locations at regular intervals. Relying on an amplicon sequencing workflow to genomically characterize the microbes present and principal components analysis to differentiate the community structure between sampling sites, surfaces and time points, researchers determined that (a) the community compositions are distinguishable by site; (b) there are also coating-specific, coating-by-site, and biocide-specific community characteristics. In a parallel longitudinal study from samples in the Chesapeake Bay, differentiation in the microbial community compositions was observed from 2–6 weeks, and differences in dispersal were observed among groups based on coating, the presence of biocide, and time in the field. From these samples, a deeper examination of the taxa present may provide new insights into the effects of different coating treatments and the mechanisms by which these communities differentially attach or disperse.

RDX is known to contaminate soils at several military training ranges presenting an environmental hazard to the local plant life [34]. Additionally, as the compound can selectively finds its way into the ground water, it represents a potential neurological and carcinogenic threat to humans [35]. One strategy to lower the mobilization of RDX in ground water is to inoculate specific aerobic biological supplements close to the soil surface. Researchers hypothesized that bioaugmenting contaminated soil with the bacterium *Rhodococcus rhodochorous* 11Y, which contains the xplAB gene cluster encoding an enzyme capable of reductively degrading RDX, can accelerate RDX breakdown. Additionally, adding compost to contaminated soil microcosms could further biostimulate the reducing environment providing an additive or synergistic effect on the rate of degradation. Through a series of controlled studies at two soil sampling locations, the effects of bioaugmentation and biostimulation were measured in soils contaminated with RDX. Measuring RDX breakdown by high performance liquid chromatography, researchers showed both bioaugmentation (adding 11Y) and biostimulation (adding compost) accelerated the breakdown of RDX. The two treatments together were faster than compost alone but, surprisingly, slower than 11Y alone. qPCR measurements of xplA gene abundance were higher for 11Y treatments, and the number of bacterial copies as measured by 16S rRNA amplification were higher for compost-treated samples. Compost drove compositional variation, with the 16S bacterial community notably changing over time while the fungal community (ITS amplification) changed to a lesser extent. 11Y and RDX treatments likewise had small but significant effects over time on bacterial community composition with no significant changes in fungal composition. Collectively, these results indicated that active bioaugmentation and biostimulation individually have the potential for aerobically degrading RDX in soil, and these treatments, especially compost stimulation, show notable shifts in bacterial community composition over time.

Microbiologically-induced corrosion (MIC) is a major problem in a variety of shipboard operations for the Navy [36]. For example, CuNi tubing in heat exchangers are sensitive to sulfide attack, and shipboard use of polluted harbor waters high in microbially-produced sulfides and low in dissolved oxygen may create conditions under which corrosion can damage these systems. Researchers used harbor water and sediment collected from Port Everglades, FL (a major cruise and commercial port), and exposed four navy-relevant metal or alloy coupons to examine the microbes associated with MIC and their community structure and corrosive potential. Sulfide levels were measured on days 7, 21, and 28 days after exposure and collected DNA for 16S amplicon sequencing from water, sediment, and biofilms on the metals at the beginning and end of the 28-day experiment. The sediment microbial population was unchanged throughout, however, the seawater microbial population had lower diversity at the end of the experiment. The sediments are enriched in Deltaproteobacteria (a clade which contains characteristic sulfide-producing bacteria) while the water samples are enriched in Alphaproteobacteria. Biofilms on the test metals showed greatest diversity in the sediment contact region, and titanium coupons had higher biodiversity overall than any of the CuNi alloys tested. To mimic operational conditions aboard ships in port, an experimental flow-loop rig was assembled to pass natural and pollutant-enriched “port” water through pipes made of the various test alloys either continuously or discontinuously for 23 days, and then biofilms were collected from these sample pipes and analyzed. Preliminary data showed higher biofilm biodiversity on plastic substrates adjacent to the metal samples than the metal samples themselves and effects of flow on biodiversity. This experimental approach will facilitate further investigation of the relationship between polluted water microbial communities and MIC in Naval water-handling systems.

The intersection of computational statistics and bioinformatic data science was explored to better understand complex biological systems and engineer practical solutions to specific challenges. Bioinformatics can be used to interpret and explore large datasets for capabilities (including de novo biological design) with high-throughput approaches beyond the scope of classical rational design. To use these tools in engineering design processes it is necessary to work not from genomes and protein databases to known functions, but rather from known or desired capabilities to existing or synthetically generated genetic sequence. One potential engineering target of computationally enhanced biocapability design is plastic/polymer recycling and valorization (recovery of plastic breakdown products as useful feedstocks for new polymer synthesis) [37], using organisms isolated from aircraft polymer surface biofilms. Nascent RNA sequencing was used to detect and quantify messenger RNA since it is produced by RNA polymerase, thus only newly made and not old/constitutive RNA sequences will be assessed at a highly resolved time course. The PCMCI (Momentary Conditional Independent Peterson-Clark) algorithm will be used to mathematically extract causal relationships from correlative data [38] and analyze time-resolved gene expression networks revealed by nascent RNASeq. This will establish causal links in the mechanisms microbes use to accomplish useful biocapabilities such as anti-biofouling or polymer bioremediation and valorization.

## Conclusion

The 8th annual TSMC symposium highlighted the diversity and potential of microbiome science occurring across the DoD research space. Broadly, the symposium highlighted three major themes: 1) the ability of microbes to serve as sentinel and surveyors of human and natural environments; 2) the developing tools that are and will allow this research to catapult forward; and 3) the ability through analysis or manipulation of microbial communities to develop and identify beneficial interventions, and increase understanding of interactions between human health and the environment.

## Supplementary Information

Additional File 1: TSMC Annual 2024 Agenda_09202024. Description of data: Meeting program of the 8th Annual TSMC Symposium containing the agenda and presentation abstracts.

## Abbreviations

AMS: Acute mountain sickness
DoD: Department of Defense
DSTL: Defense Science and Technology Laboratory
IVA: Tetra-acylated lipid A
MIC: Microbiologically-induced corrosion
PCMCI: Momentary Conditional Independent Peterson-Clark
PTSD: Post-Traumatic Stress Disorder
RDX: Royal Demolition Explosive
TBI: Traumatic brain injury
TSMC: Tri-Service Microbiome Consortium
