# Supplementary material for: Meeting report of the eight annual Tri-Service Microbiome Consortium Symposium
Source: BMC Proc. 2025 Dec 23;20(Suppl 2):2. doi: 10.1186/s12919-025-00355-z (PMC12723844; doi:10.1186/s12919-025-00355-z)
Supplement: Supplementary file 1 — Additional File 1: TSMC Annual 2024 Agenda_09202024. Description of data: Meeting program of the 8th Annual TSMC Symposium containing the agenda and presentation abstracts. [file 12919_2025_355_MOESM1_ESM.pdf]

TSMC 2024 Annual Program

Hybrid Meeting, The Antlers, Colorado Springs, CO
25-26 September 2024

TSMC
Tri-Service Microbiome Consortium

Welcome to TSMC 2024!

On behalf of the Tri-Service Microbiome Consortium (TSMC), we welcome you to the Office of the Undersecretary of Defense (Research & Engineering), Biotechnology Community of Interest, 8th Annual TSMC Meeting: TSMC 2024! We are looking forward to a two day feast of microbiome presentations and discussions from DoD researchers and our Government, Industry, and Academic partners.

The TSMC is a forum for DoD microbiome researchers to communicate ongoing research within the Army, Navy, and Air Force to identify research and capability gaps and coordinate research, while leveraging capabilities and resources. The annual TSMC meeting is designed to enable information sharing between DoD scientists and leaders in the field of microbiome science, thereby keeping DoD consortium members informed of the latest advances within the microbiome community and facilitating the development of new collaborative research opportunities. We publish meeting reports from our Annual Meetings, so please check them out if you are interested in learning more about microbiome research within the DoD.

We encourage you all to take advantage of the interactive features of our hybrid event as much as possible to make TSMC 2024 as vibrant as usual. We also hope to see you at our no-host social on Wednesday evening!

We hope you find TSMC 2024 informative and useful.

Let the symbioses begin!

Michael Goodson | Chair, TSMC
711th Human Performance Wing
Air Force Research Laboratory,
Wright-Patterson Air Force Base, OH 45433

Dasha Leary | Vice-Chair, TSMC
Naval Research Laboratory
Washington, DC 20375

Acknowledgements

We would like to thank all of those who have contributed to the planning and success of TSMC2024! We feel this long and distinguished list conveys the interest and importance of this meeting to the Department of Defense and to the microbiome field in general.

Specifically, we would like to thank:

- User Community, Special Session Speakers, Oral Presenters, Poster Presenters, and the TSMC 2024 Participants for their interest and commitment.
- TSMC 2024 Annual Meeting Planning Committee for their dedication and enthusiasm: Dr. Michael Goodson (TSMC Chair), Dr. Dasha Leary (TSMC Vice Chair); Dr. Richard Agans; Dr. Robyn Barbato; Dr. Rasha Hammamieh; Dr. Phil Karl; Dr. Bob Kokoska; LCDR Neal McNeal; Mr. Ken Racicot, Mr. Jason Soares, Dr. Blake Stamps, Dr. Charlie Sweet, and Dr. Jordan Steel.
- Session Chairs for moderating their sessions: Dr. Richard Agans; Dr. Robyn Barbato; Ms. Stacey Doherty; Dr. Bob Kokoska; Dr. Zach Liechty; LCDR Neal McNeal; Mr. Ken Racicot; Mr. Jason Soares; Dr. Blake Stamps; Dr. Jordan Steel; Dr. Charlie Sweet; Ms. Sara Tuck; and Mr. Jordan Whitman
- Dr. Melissa Kardish for coordinating the 8th Annual TSMC Symposium meeting report
- The Office of the Undersecretary of Defense (Research & Engineering), Biotechnology Community of Interest, Chaired by Dr. Peter Emanuel, for their generous support of the meeting, and Mr. Jay Haugen and Ms. Jill McQuade for their valuable assistance.
- And finally, we would especially like to thank Ms. Lorrie Strausbaugh, Ms. Kristen Fritz, Drs. Veeraraghavan Sundar and Stephaney Shanks, with their teams at BlueHalo, for handling the logistics surrounding TSMC2024 with such patience and enthusiasm.

If you are interested in learning more about the TSMC, please reach out to your TSMC working group representative:

Dr. Michael Goodson
Chair (AFRL)

Dr. Dasha Leary
Vice-Chair (NRL)

Army

Dr. Robyn Barbato (ERDC-CRREL)
MAJ Blair Dancy, Former Vice-Chair (44 MED)
Dr. Rasha Hammamieh (WRAIR, OWP co-chair)
Dr. J. Philip Karl (USARIEM)
Dr. Robert Kokoska (ARO)
Mr. Kenneth Racicot (DEVCOM SC)
Mr. Jason Soares (DEVCOM SC)

Air Force

Dr. Richard Agans (AFRL)
Dr. Nancy Kelley-Loughnane (AFRL, EWS co-chair)
Dr. Camilla Mauzy (AFRL)
Dr. Blake Stamps (AFRL)

Navy

Dr. Sophie Colston (NRL)
Dr. Kristy Hentchel (ONR)
Dr. Melissa Kardish (NRL)
LCDR Neal McNeal (NSMRL)
Dr. Gary Vora (NRL)

Event Resources

- TSMC Annual Meeting 2024 Website
- Download the latest version of Zoom here
- Click here to join Day 1 Meeting Link
- Click here to join Day 2 Meeting Link

Past TSMC Meeting Resources

TSMC ANNUAL MEETING REPORTS

- 1st Annual Meeting
- 2nd Annual Meeting
- 3rd Annual Meeting
- 4th Annual Meeting
- 5th Annual Meeting Part 1: Current advances in microbiome sciences within the US Department of Defense—part 1: microbiomes for human health and performance

[doi: 10.1136/military-2022-002307, Colston SM, et al. BMJ Mil Health Published Online First: 15 June 2023]

- 5th Annual Meeting Part 2: Current advances in microbiome sciences within the US Department of Defense—part 2: microbiomes for human health and performance

[doi: 10.1136/military-2022-002308, Colston SM, et al. BMJ Mil Health Published Online First: 18 June 2023]

- 6th Annual Meeting
- 7th Annual Meeting: Report available soon in 'BMC Proceedings'

OTHER TSMC PUBLICATIONS

- Evaluation of Probiotics for Warfighter Health and Performance
[doi: 10.3389/fnut.2020.00070, Agans, Richard T., et al. " Frontiers in Nutrition 7 (2020): 70]

- Gut Microbiota-Targeted Interventions for Reducing the Incidence, Duration and Severity of Respiratory Tract Infections in Healthy Non-Elderly Adults
[doi:10.1093/milmed/usaa261, Karl, J. Philip, Military Medicine 186.3-4 (2021): e310-e318]

- Orally ingested probiotics, prebiotics, and synbiotics as countermeasures for respiratory tract infections in nonelderly adults: a systematic review and metanalysis [doi:10.1093/advances/nmac086, Coleman, Julie L., et al., Advances in Nutrition 13.6 (2022): 2277-2295]

- Orally ingested probiotics, prebiotics, and synbiotics as countermeasures for gastrointestinal tract infections in nonelderly adults: a systematic review and metanalysis
[doi:10.1016/j.advnut.2023.02.002, Fagnant, Heather S., et al., Advances in Nutrition (2023)]

Agenda Outline – All Times MST

TUESDAY, 24 SEPT

Time	
**1600-1800**	**Optional Tour of USAFA (POC – Dr. Jordan Steel)**

WEDNESDAY, 25 SEPT – DAY 1 MORNING

**Time** (MST)	
**0730-0830**	**Check-in/Login/Morning Social**
**0830-0850**	**Opening Remarks & TSMC overview -** Dr. Michael Goodson, Chair, AFRL **and** Dr. Dasha Leary, Vice-Chair, NRL
**0850-0900**	**USAFA Welcome –** Dr. Jordan Steel, USAFA
**0900-0930**	**OUSD (R&E) Biotechnology Overview and Biotech Community of Interest Overview -** Dr. Peter Emanuel, DEVCOM CBC, Steering Committee Chair

Special Session – Digital Data Backbone Environments

**Time** (MST)	**Topic**	**Presenter(s)**
**0930-0950**	A Tri-Service Data Backbone to Enable Microbiome Science	Dr. Blake Stamps (AFRL) & Dr. Judson Hervey (NRL)
**0950-1000**	Digital Data Backbone Q&A	
**1000-1030**	**Morning Break**	

Technical Session #1- Surveillance

Chairs: Dr. Jordan Steel (USAFA), Dr. Robyn Barbato (CRREL)

**Time** (MST)	**Abstract Title**	**Presenter(s)**
**1030-1050**	Microbial profiling of clinical specimens to verify pathogen panels	Ms. Monica Christian (AFRL) - Virtual
**1050-1110**	Differential toxicity of classes of chemical pollutants on relative abundance of microbiota	Dr. Seid Muhie (WRAIR) - Virtual
**1110-1130**	Microbial community dynamics and substrate utilization in a permafrost-active layer soil mixing experiment	Ms. Stacey Doherty (CRREL)

**1130-1150**	Wastewater surveillance of viral and bacterial pathogens	MAJ William Kowallis (DCPH-Aberdeen)
**1150-1205**	Session Panel Q&A	
**1205-1330**	**Lunch Break**	

WEDNESDAY, 25 SEPT – DAY 1 AFTERNOON

Technical Session #2 - Health and Performance 1

Chairs: LCDR Neal McNeal (NSMRL), Dr. Zach Liechty (AFRL)

**Time** (MST)	**Session**	**Presenter(s)**
**1330-1350**	Acute Trauma-Induced Microbiome Changes are Sex-Dependent in Rats	CPT Matthew Rusling (WRAIR) – Virtual
**1350-1410**	The Pre-Ascent Gut Microbiome Associates with Acute Mountain Sickness Severity During High Altitude Sojourn	Mr. Devin Connolly (USARIEM)
**1410-1430**	Understanding the microbiome of military personnel	Dr. Iain Templeman (UK DSTL)
**1430-1450**	Traumatic brain injury and the gut microbiome	Dr. Lisa Brenner (VA MIRECC)
**1450-1505**	Session Panel Q&A	
**1505-1530**	**Break**	
**1530-1730**	Poster Session	Chairs: Mr. Jordan Whitman, Mr. Jason Soares (DEVCOM SC)
**1800-1930**	**'No Host' Social at Hotel Bar**	

THURSDAY, 26 SEPT – DAY 2 MORNING

Time (MST)	Session	Presenter(s)
0730-0820	Morning Social	
0820-0830	Welcome - Dr. Michael Goodson, Chair, AFRL and Dr. Dasha Leary, Vice-Chair, NRL	

Special Session - DARPA and DTRA Microbiome Programs

0830-0850	DARPA BTO	Dr. Tiffany Prest
0850-0910	DTRA	Dr. Lalena Wallace
0910-0920	DARPA and DTRA Microbiome Programs Session Q&A	

Special Session - User Community

0920-0940	Surg. Cdr. Alistair Morris, Royal Navy / UK Exchange Officer, NSMRL	
0940-1000	CMSgt. Eric Peterson, USAF	
1000-1010	User Community Session Q&A	
1010-1040	Break	

Technical Session #3 – Enablers

Chair: Dr. Richard Agans (USAFSAM), Ms. Stacey Doherty (CRREL)

Time (MST)	Session	Presenter(s)
1040-1100	Bioprospecting Workflows with the Whole HPC Toolkit	Dr. Margaret Hurley (DEVCOM ARL) – Virtual
1100-1120	Quality Assessment and Quantifying Variations Across 16S rRNA Sequence Data	Dr. Christopher Stamper (VA MIRECC)
1120-1140	Multi-Input Deep Neural Networks for Characterizing Metagenomic Profiles and Clinical Assessments in Deployed Environments	Dr. Camilo Valdes (LLNL)
1140-1200	The ArtGut in vitro system	Dr. Todd Thorsen (MITLL)
1200-1215	**Session Panel Q&A**	
1215-1330	Lunch	

Technical Session #4 – Remediation

Chairs: Dr. Bob Kokoska (ARO), Dr. Charlie Sweet (USNA)

Time (MST)	Session	Presenter(s)
**1330-1350**	Thinking globally about marine microbial communities on future U.S. Navy coatings	Dr. Melissa Kardish (NRL)
**1350-1410**	Biostimulation of RDX degradation in two rangeland soils	Dr. Chris Baker (CRREL)
**1410-1430**	Exploring the Corrosion Microbiome of Copper-Nickel Alloys in Natural Seawater	Dr. Rachel Mugge (NRL)
**1430-1450**	Characterizing polymer valorization by microbial fungi isolated from aircraft using time-series nascent RNA-Seq analysis	Dr. Dominique Wagner (AFRL)
**1450-1505**	**Session Panel Q&A**	
**1505-1530**	**Break**	

Technical Session #5 - Health and Performance 2

Chair: Mr. Ken Racicot (DEVCOM SC), Ms. Sara Tuck (NRL)

Time (MST)	Session	Presenter(s)
**1530-1550**	Microbiome Dynamics in Swine Wound Model	Dr. Aarti Gautam (WRAIR) – Virtual Presenter
**1550-1610**	Shigellosis results in persistent changes to the gut microbiome	Dr. Zachary Liechty (AFRL)
**1610-1630**	Impact of melanized bacteria on the intestinal homeostasis and microbiome	Dr. Zheng Wang (NRL)
**1630-1650**	Alcohol influences gut microbiome: results and implications for United States military members and Veterans	Dr. Andrew Hoisington (VA MIRECC)
**1650-1705**	**Session Panel Q&A**	
**1705-1715**	**Closing Remarks** - Dr. Michael Goodson, Chair, AFRL and Dr. Dasha Leary, Vice-Chair, NRL	

Virtual Meeting Information

The virtual meeting will be hosted on the FEDRAMP-Approved ZoomGov Platform.

Topic: TSMC Annual Meeting

Time: Eastern Time (US and Canada)

Sep 25, 2024 07:00 AM

Sep 26, 2024 07:00 AM

Please download and import the following iCalendar (.ics) files to your calendar system.

Daily:
https://uesincdayton.zoomgov.com/meeting/vJIscOGvrjsqHuKJ0hDCkOLmPkqklOaQliw/ics?icsToken=98tyKuiurzwiHdSVsB3BeI86FaPKaOnrkWBmv4h3zBfBICt8Tg7GPfNJPp1-IdTZ

Join ZoomGov Meeting

https://uesincdayton.zoomgov.com/j/1617870004?pwd=184CGhbchkiY8oIuErqOe2k6b6oJrW.1

Meeting ID: 161 787 0004

Passcode: 372635

One tap mobile

+16692545252,,1617870004# US (San Jose)

+16468287666,,1617870004# US (New York)

Dial by your location

- +1 669 254 5252 US (San Jose)
- +1 646 828 7666 US (New York)
- +1 646 964 1167 US (US Spanish Line)
- +1 669 216 1590 US (San Jose)
- +1 415 449 4000 US (US Spanish Line)
- +1 551 285 1373 US (New Jersey)

Meeting ID: 161 787 0004

Find your local number: https://uesincdayton.zoomgov.com/u/a74sOhDfg

Table of Contents

Technical Session #1: Surveillance 10

Wastewater surveillance of viral and bacterial pathogens at the US Naval Academy using qPCR, targeted sequencing, and whole genome shotgun sequencing: A concerted effort with ongoing USNA respiratory surveillance efforts. 10

Microbial profiling of clinical specimens to verify negative pathogen panels 10

Microbial community dynamics and substrate utilization in a permafrost-active layer soil mixing experiment 11

Differential toxicity of classes of chemical pollutants on relative abundance of microbiota: a shotgun metagenomic study 12

Technical Session #2: Health and Performance 1 12

Traumatic brain injury and the gut microbiome: analyses from animal & human acute and post-acute studies................ 12

The Pre-Ascent Gut Microbiome Associates with Acute Mountain Sickness Severity During High Altitude Sojourn............ 13

Understanding the microbiome of military personnel.. 14

Acute Trauma-Induced Microbiome Changes are Sex-Dependent in Rats 14

Technical Session #3: Enablers 15

Bioprospecting Workflows with the Whole HPC Toolkit.. 15

Quality Assessment and Quantifying Variation Across 16S rRNA Sequence Data – From Sample Collection to Sequencing in Human Microbiome Studies.. 15

Multi-Input Deep Neural Networks for Characterizing Metagenomic Profiles and Clinical Assessments in Deployed Environments 16

The ArtGut *in vitro* system 16

Technical Session #4: Remediation 17

Thinking globally about marine microbial communities on future U.S. Navy coatings... 17

Biostimulation of RDX degradation in two rangeland soils 18

Exploring the Corrosion Microbiome of Copper-Nickel Alloys in Natural Seawater 18

Characterizing polymer valorization by microbial fungi isolated from aircraft using time-series nascent RNA-Seq analysis 19

Technical Session #5: Health and Performance 2 19

Alcohol influences gut microbiome: results and implications for United States military members and Veterans................ 19

Shigellosis results in persistent changes to the gut microbiome 20

Microbiome Dynamics in Swine Wound Model: Evaluating Topical Noneuphoric Phytocannabinoid Elixir 14 Against Standard Silver Dressing.. 21

Impact of melanized bacteria on the intestinal homeostasis and microbiome: potential probiotic for treatment of radiation injury and inflammation diseases 22

Posters 23

Technical Session #1: Surveillance

WASTEWATER SURVEILLANCE OF VIRAL AND BACTERIAL PATHOGENS AT THE US NAVAL ACADEMY USING QPCR, TARGETED SEQUENCING, AND WHOLE GENOME SHOTGUN SEQUENCING: A CONCERTED EFFORT WITH ONGOING USNA RESPIRATORY SURVEILLANCE EFFORTS

Millward, GG (Defense Centers Public Health – Aberdeen, Aberdeen Proving Ground, MD); Kok, CR (Lawrence Livermore National Laboratory, Livermore, CA); Colombo, R (Infectious Disease Research Collaboration Program; Henry M. Jackson Foundation for the Advancement of Military Medicine); Be, NA (Lawrence Livermore National Laboratory, Livermore, CA); Kowallis, WJ (Defense Centers Public Health – Aberdeen, Aberdeen Proving Ground, MD); Agans, RT (U.S. Air Force School of Aerospace Medicine, Dayton, OH)

US Service Members are particularly susceptible to transmissible pathogens due to increased exposure in congregate settings. As such, outbreaks within barracks, training and medical facilities, or transport vehicles can quickly lead to decreased operational readiness and reduced force health protection. Wastewater Surveillance proved to be invaluable during the recent COVID-19 pandemic in tracking community wide emergence of variants and other respiratory viruses of concern. The utility and popularity of wastewater surveillance as a cost-effective, scalable, and non-invasive approach to assessing community public health has led to a CDC national wastewater surveillance network to gather wastewater data from across the United States. Furthermore, presence of viral pathogens within wastewater have been detected before recorded clinical cases. Currently no concerted efforts exist within DoD CONUS sites to pair clinical and wastewater surveillance, particularly with the goals of establishing and optimizing infrastructure, multi-threat detection and characterization via NGS;nor has there been focus on co-occurrence of respiratory viral and/or bacterial pathogens. Further, no DoD site has validated respiratory pathogen WWS against an intensively clinically surveilled location which undertakes sequencing of major respiratory pathogens through clinical case virological surveillance. USAFSAM, DCPH-A and Lawrence Livermore National Laboratory are working with the IDCRP to profile respiratory viruses and bacterial virulence markers from wastewater collected at and around the US Naval Academy reconciled against medically attended acute respiratory infections over a one year period. The study aims to 1) augment ongoing case-based respiratory pathogen surveillance with wastewater monitoring, via established qPCR methods to identify increases in respiratory pathogens (including coronaviruses, influenza, rhino/enteroviruses, etc.) before increases in medically attended case and congregate setting outbreaks at the USNA, 2) characterize known and novel respiratory viruses via hybrid capture whole genome sequencing, 3) determine occurrence of bacterial pathogens, 4) assess seasonal fluctuations among pathogen signals, and 5) assess the longitudinal relationship between clinical and wastewater data to enhance recommendations for decision making and predictive machine learning models.

MICROBIAL PROFILING OF CLINICAL SPECIMENS TO VERIFY NEGATIVE PATHOGEN PANELS

Christian, MC ; Hanson, JF ; Kramer, DL ; Capati, RH ; Agans, RT - U.S. Air Force School of Aerospace Medicine, Dayton, OH;Venesco LLC, Chantilly, VA.

Respiratory and gastrointestinal infectious diseases present significant burdens to force health protection efforts and overall unit readiness. Respiratory infections are responsible for upwards of 95,000 lost duty days each year impacting upwards of 600,000 active duty members. Gastrointestinal infections remain a significant burden within the DoD. Approximately 30% of Service Members traveling >1 month experience debilitation leaving them incapable of carrying out duties and activities. Service Members experiencing symptoms submit clinical specimens to be sent to reference laboratories for detection and identification of causative agents. The DCPH-Dayton (DCPH-D) Epidemiology Laboratory is the DoD reference lab for clinical diagnostics and reporting. They receive >12,000 specimens yearly from medical facilities across the globe. Approximately 67% fail to identify

an agent to explain patient symptoms. DCPH-D's Applied Technology and Genomics lab is proficient in several molecular and biochemical techniques and often serves to verify and develop assays with direct application back in the Epidemiology group. Specifically, metagenomic shotgun sequencing profiling of microbiota consortia can reveal previously undetected links to morbidities and clinical observations. The current effort seeks to (1) determine microbial consortia profiles in respiratory and fecal samples between those with positive and negative pathogen detections and (2) as molecular genetic data can reveal sensitive information about population demographics and health status, this work will determine whether geographic, gender, and other patient metadata can be presumed from obtained data. DCPH-D is currently collecting, and processing, 800 samples across respiratory and fecal submissions (200 each, positive/negative). Total nucleic acids are extracted/purified through Maxwell RSC kits, RNA is reverse-transcribed to cDNA and metagenomics performed using Illumina NovaSeq, resulting metagenome reads are then processed in custom workflows to remove human reads, generate genome assemblies, and annotate individual consortia members, followed by bioinformatics analyses between consortia and aggregated sample metadata. Microbial communities contain complex consortia of different bacterial, viral, parasitic, and fungal members that communicate bidirectionally, and their disruption can result in significant negative impacts to humans. Understanding the complexities of microbiota composition and communication resulting from infectious organisms through microbial profiling will help determine if previously identified negative specimens correlate to those of known positive specimens.

MICROBIAL COMMUNITY DYNAMICS AND SUBSTRATE UTILIZATION IN A PERMAFROST-ACTIVE LAYER SOIL MIXING EXPERIMENT

Stacey Doherty, Dr. Alison Thurston, Dr. Robyn Barbato
United States Army, Engineer Research Development Center, Cold Regions Research and Engineering Laboratory, Hanover, NH

Permafrost soils in the Earth's cold regions are experiencing rapid degradation due to climate warming which poses unique challenges to Army operations in high latitude regions. Microbial communities entrained in permafrost undergo significant compositional and functional shifts as soils thaw. Dispersal of microbial communities from the seasonally-thawed active layer soil into newly thawed permafrost may influence community assembly and increase carbon release from soils. We conducted a laboratory soil mixing experiment to understand how carbon utilization and microbial community structure are affected by the mixing of active layer and permafrost soils when terrain thaws. We hypothesized that when active layer soil was added to permafrost, carbon utilization would increase but when permafrost was added to active layer soil, there would be little effect on carbon utilization because active layer soils contain greater biomass and microbial diversity compared to permafrost. Active layer soil and permafrost collected from two sites in interior Alaska were mixed in five different ratios and incubated for 100 days at 10°C to reflect current maximum surface soil temperatures at these sites. Heterotrophic respiration was measured throughout the incubation to quantify soil activity. Respiration rates were highest in the 100% active layer soils, averaging 19.8 µg C-CO2 g-1 dry soil d-1 across both sites, and decreased linearly as the ratio of permafrost increased. Biolog Ecoplates were used to survey metabolic patterns of the mixed communities by measuring carbon substrate utilization. Mixing of the two soil layers resulted in utilization of a more diverse group of carbon substrates compared to active layer or permafrost alone. Additionally, combining active layer and permafrost soils increased microbial diversity and resulted in communities that resembled those from the active layer when soils were mixed in equal ratios. Microbial communities of the experimentally mixed soils did not resemble those collected from the active layer-permafrost transition zone. Understanding the effects of active layer and permafrost soil mixing could improve

predictions of carbon-climate feedbacks and domain awareness as permafrost thaws in these regions.

DIFFERENTIAL TOXICITY OF CLASSES OF CHEMICAL POLLUTANTS ON RELATIVE ABUNDANCE OF MICROBIOTA: A SHOTGUN METAGENOMIC STUDY

Seid Muhie[1,2], Aarti Gautam[1], Erik Mylroie[3], Bintu Sowe[1], Ross Campbell[1], Rasha Hammamieh[1], Edward Perkins[3], Natalia Vinas[3] and Rasha Hammamieh[1]**

[1]Medical Readiness Systems Biology, CMPN, Walter Reed Army Institute of Research, Silver Spring, MD.

[2] The Geneva Foundation, Tacoma, WA.

[3] U.S. Army Engineer Research and Development Center Environmental Laboratory, Vicksburg, MS.

Toxicity due to environmental pollutants are known to adversely affect the biodiversity of living organisms. Particularly, chemical pollutants tend to accumulate in the environment over time, distorting the natural balance of biodiversity of microbial populations. Here, we collected sediment and water samples from eight sites within the Great Lakes (USA). Samples were characterized using shotgun metagenomic sequencing and chemical analysis for more than 200 chemicals belonging to ~ 16 broad classes of chemicals (pesticides, industrial products, personal care products and pharmaceuticals). We carried out integrative and differential comparative and correlation analyses on the bimodal datasets. Microbiota density (as approximated by adjusted total counts of sequence reads) decreased with an increased total concentration of chemical pollutants. Protozoan, metazoan, and fungal populations were negatively correlated with concentrations of chemical pollutants whereas some bacterial (proteobacteria) and archaeal populations were positively correlated with increasing concentrations of chemical pollutants. As expected, concentrations of chemical pollutants from sediment samples were at much higher concentration and had larger dynamic range compared to concentrations of the same class of chemicals in water samples. Among other factors, differential concentration of pollutants may be the reason why we see that some bacterial, metazoan, and protozoan population showed detectable abundance only at certain sites or sample types (water or sediment). Our preliminary finding shows that microbial diversity could potentially be correlated with the type and concentration of chemical pollutants, and can be used as proxy-markers to assess type and level of the corresponding toxicity due to chemical contaminants.

Technical Session #2: Health and Performance 1

TRAUMATIC BRAIN INJURY AND THE GUT MICROBIOME: ANALYSES FROM ANIMAL & HUMAN ACUTE AND POST-ACUTE STUDIES

Andrew J. Hoisington, Department of Veterans Affairs; Christopher E. Stamper, Department of Veterans Affairs; Kelly A. Stearns-Yoder, Department of Veterans Affairs; Teodor T. Postolache, Department of Veterans Affairs; Christopher A. Lowry, University of Colorado, Boulder; Tara Cominski, Department of Veterans Affairs; Kevin Beck, Department of Veterans Affairs & Lisa A. Brenner, Department of Veterans Affairs

Traumatic brain injury (TBI) has been described as one of the signature wounds of Operation Enduring Freedom/Operation Iraqi Freedom. Acute TBI exerts damage to the brain through an external force that activates a cerebral inflammatory response and/or changes to the gut microbiome. Few treatment options exist for those with TBI, therefore a better understanding of biological responses connected to physical/mental health outcomes is needed. We report on three

distinct studies: a preclinical animal model sampling up to 30 days post-injury;a clinical mild TBI investigation with data collected between 48 hours to12 months post-injury;and a post-acute TBI study. Briefly, in the preclinical mild TBI study, we observed sex-specific differences for rats in gut microbiome responses to injury, with females showing more taxonomic differences over time, that included short chain fatty acid producing genera associated with immunoregulatory processes, as compared to males. In the post-acute study, post-injury individuals had a significantly divergent microbial community, as compared to baseline. A rapid reduction in abundance, with no long-term recovery, of the potentially anti-inflammatory genus Akkermansia partially explained the trend. Finally, in the post-acute human study, we observed no significant microbial community differences between individuals with moderate/severe TBI and no differentially abundant taxa, as compared to individuals without a history of such injury. The negative result might be explained by the mean 26 years between injury and sampling, as well as co-occurring conditions. Studies are needed to identify factors driving microbiome changes post-TBI to target interventions aimed at ameliorating negative health outcomes.

THE PRE-ASCENT GUT MICROBIOME ASSOCIATES WITH ACUTE MOUNTAIN SICKNESS SEVERITY DURING HIGH ALTITUDE SOJOURN

Devin A. Connolly[1,2], Peter S. Figueiredo[1], Janet E. Staab[1], Steven D. Landspurg[1,2], Beth A. Beidleman[1], J. Philip Karl[1]
[1]U.S. Army Research Institute of Environmental Medicine, Natick, MA
[2]Oak Ridge Institute for Science and Education, Oak Ridge, TN

BACKGROUND
Acute Mountain Sickness (AMS) is a physiological condition resulting from the low partial pressure of atmospheric oxygen at high altitude (>2,500 m above sea level). AMS symptoms range in severity from nuisance to life-threatening and demonstrate wide unexplained inter-individual variability. The gut microbiome may influence physiologic responses to high altitude that could contribute to the development and severity of AMS.

PURPOSE: This study aimed to identify gut microbiome features measured before ascent to high altitude that are associated with AMS severity at altitude.

METHODS: Seventy-five healthy soldiers (26±5yr, 11% female) were tested at their baseline residence (BLR; ≤1,200 m) and during a 4-day high altitude sojourn (HA; 3,600 m). Gut microbiome composition and functional pathways were measured in two fecal samples collected at BLR using shotgun metagenomics sequencing. Incidence and severity of AMS were assessed multiple times daily at HA using the Environmental Symptoms Questionnaire. Peak AMS-Cerebral Factor score (AMS-C) during the sojourn was used to classify participants as experiencing no (<0.7) , mild (≥0.7 and <1.53) , or moderate/severe AMS (≥1.53).

RESULTS: Incidence of moderate/severe AMS and mild AMS was 19% and 24%, respectively. AMS responder groups did not differ by sex, age, body-mass index (BMI) or race (P>0.05). Between-group differences in species-level bacterial composition were driven by the moderate/severe-AMS group, which demonstrated lower species richness (P=0.02) and differences in β-diversity (P=0.007) relative to the mild-AMS and no-AMS groups. Differential abundance analyses identified 5 species that differed in relative abundance across groups (q<0.25): Alistipes dispar, Bacteroides sp. CACC 737, Bacteroides helcogenes, Hoylesella enoeca, and Eubacterium sp. MSJ-33.

CONCLUSIONS : Gut microbiome features measured before ascent are associated with AMS severity

during high altitude sojourn. Ongoing analyses aim to identify bacterial gene pathways associated with AMS severity to elucidate potential underlying mechanisms linking the gut microbiome to AMS severity.

Disclaimer: This work was supported by the Assistant Secretary of Defense for Health Affairs, through the Peer Reviewed Medical Research Program (PRMRP) under Award Number CDMRP-22-PR220729 and the Military Operational Medicine Research Program. Opinions, interpretations, conclusions, and recommendations are those of the author and are not necessarily endorsed by the Department of Defense..

UNDERSTANDING THE MICROBIOME OF MILITARY PERSONNEL

Sarah Harding and Iain Templeman

The human microbiome has a role in many bodily functions, including the production of bioactive metabolites, regulation of the immune response and the digestion of food. Evidence suggests that specific components of the gut microbiome may mediate aspects of health and performance, including susceptibility to disease, cognitive function following sleep deprivation and endurance capacity. However, despite these effects, the composition and activity of the human microbiome can be modified by external factors such as diet and antibiotic treatment. This is particularly pertinent in relation to defence personnel, who are also subject to a range of environmental exposures with largely unknown effects on the human microbiome, such as extremes of temperature, altitude, and disrupted sleep, which by extension could impact both their health and level of performance. This in turn can impact operational readiness and effectiveness, particularly when duty days are lost due to illness or reduced performance. Both diarrhoeal disease and respiratory infections cause significant problems, particularly on deployments. A better understanding of the composition of these microbiomes and how they respond and adapt to military relevant stressors may therefore enable interventions to be developed and delivered, with the aim of protecting health and ultimately performance.

© Crown copyright (2024), Dstl. This material is licensed under the terms of the Open Government Licence except where otherwise stated. To view this licence, visit http://www.nationalarchives.gov.uk/doc/open-government-licence/version/3 or write to the Information Policy Team, The National Archives, Kew, London TW9 4DU, or email: psi@nationalarchives.gov.uk

ACUTE TRAUMA-INDUCED MICROBIOME CHANGES ARE SEX-DEPENDENT IN RATS

Matthew R. Rusling (1), James C. DeMar (1), Rayan M. Dennett (1), Allison V. Hoke (1), Nela I. Crespo Rosales (1), Stephen R. Butler (1), Aurian Naderi (1), Abraham J. Han (1), Kollin T. Sharpes (1), Larry P. Simmons (1), Emily M. Scott (1), Rachel M. Taylor (1), Emily G. Lowery-Gionta (1), Aarti Gautam (1), and Rasha Hammamieh (1).
Affiliations: *(1) Medical Readiness Systems Biology Branch, Walter Reed Army Institute of Research, Silver Spring, MD. (2) Behavioral Biology Branch / Performance Assessment and Chemical Evaluation Lab, Walter Reed Army Institute of Research, Silver Spring, MD.*

BACKGROUND: Evidence is emerging that development of Post-Traumatic Stress Disorder is, at least in part, dependent upon microbiome state and post-stress remodeling.

OBJECTIVES: In an animal model, determine whether changes to the gut microbiome occur acutely after stress, and if any compositional shifts are sex dependent.

METHODS: Male and female adult rats were assigned to control or stress exposure groups, using a series of stressors: predators, inescapable foot shock, and underwater submersion. Fecal pellets were collected prior to and 24 hours post-exposure. Behavior was determined using elevated plus maze, open field, and acoustic startle response tests. Extracted fecal DNA was 16S V4 amplified sequenced. Data were processed with dada2 and QIIME2 before exporting to R.

RESULTS: Alpha diversity increased significantly only in the male stress group. Beta diversity was significantly affected by time and sex, but not by stress. Compositionally, most features were non-normally distributed. Across groups, female microbiomes were strongly populated with A. muciniphila. There was not a direct time effect, but across sexes there was a significant time x exposure interaction for Gammaproteobacteria, Bacilli, and Bacteroidia subclasses. Within group pre-post differences were observed only following stress and were sex-dependent even at the phyla level. Bacteria biochemical pathways were also significantly affected by stress and different between sexes. Behavioral tests revealed significant effects of sex and stress.

CONCLUSIONS: Microbiome remodeling occurs almost immediately after stress exposure, with males and females displaying different microbiotic responses. Our findings have implications for current efforts to develop microbiome targeted treatments for mitigating stress.

Technical Session #3: Enablers

BIOPROSPECTING WORKFLOWS WITH THE WHOLE HPC TOOLKIT

Rebecca Renberg - DEVCOM Army Research Laboratory; Margaret M. Hurley - DEVCOM Army Research Laboratory

Here we discuss the inhouse workflow, experimental and computational, developed to collect, screen, propagate, characterize and analyze microbial communities and isolated organisms. Refinement of this process and improved throughput represents a necessary first step in characterizing and understanding the microbial communities found in our bioprospecting studies.

QUALITY ASSESSMENT AND QUANTIFYING VARIATION ACROSS 16S RRNA SEQUENCE DATA – FROM SAMPLE COLLECTION TO SEQUENCING IN HUMAN MICROBIOME STUDIES

Christopher A. Stamper [1,2], Andrew J. Hoisington [1-3], Lisa A. Brenner [1-2,4,5]
[1] Department of Physical Medicine & Rehabilitation, University of Colorado Anschutz Medical Campus, Aurora, CO, USA
[2] VA Rocky Mountain Mental Illness Research Education and Clinical Center (MIRECC), Rocky Mountain Regional VA Medical Center (RMRVAMC), Aurora, CO, USA
[3] Department of Systems Engineering & Management, Air Force Institute of Technology, Wright-Patterson AFB, OH, USA
[4] Department of Psychiatry, University of Colorado Anschutz Medical Campus, Aurora, CO, USA
[5] Center for Neuroscience, University of Colorado Anschutz Medical Campus, Aurora, CO, USA

A myriad of connections between the microbiome, physical health, and mental health have been discovered that are relevant to war fighters both during and after their military service. Rapid growth of microbiome sequencing technology did not prioritize quality assessment as a primary focus impacting reproducibility. Since 2016, our research team has sequenced ~6,000 fecal, oral, and skin

16S rRNA microbiome participant samples plus over 180 technical replicates (e.g. commercial mock community and positive control samples). Here we present the sequencing results in variation in the following: sampling collection techniques, shipping at ambient conditions, DNA concentration, sequencing runs. A direct comparison between stabilized fecal samples and unstabilized swab samples from the same participants revealed sampling methods significantly influenced microbial community results. These differences persisted even if swabs samples were frozen within 36 hours of collection. Shipping swabs at ambient conditions resulted in increases in Gammaproteobacteria and Bacilli that correlated with transit days. Technical replicate data from 18 sequencing runs revealed limitations in accuracy while precision was robust for mock community and human positive control samples. The sample type with the highest technical replication also had the highest DNA concentrations. Importantly, technical variation in the microbial community due to sequencing run was significantly less than biological variation. Overall, this research highlights the importance of the continued pursuit of standardization in microbiome research to limit technical noise and increase the reliability when combining multiple studies to benefit military members and Veterans.

MULTI-INPUT DEEP NEURAL NETWORKS FOR CHARACTERIZING METAGENOMIC PROFILES AND CLINICAL ASSESSMENTS IN DEPLOYED ENVIRONMENTS

Camilo Valdes, Lawrence Livermore National Laboratory, Andre Goncalves, Lawrence Livermore National Laboratory, Car Reen Kok, Lawrence Livermore National Laboratory, Joanna Halford, Naval Submarine Medical Research Laboratory, Michael Goodson, Air Force Research Laboratory, Nicholas Be, Lawrence Livermore National Laboratory, LCDR Neal McNeal, Naval Submarine Medical Research Laboratory

There are many aspects of military deployment that exert unique pressures on the human microbiome, and this is reflected in the microbiome samples of military personnel. However, it can be challenging to characterize these samples in the context of clinical assessments of health states and performance metrics, which is why this study focused on deep neural networks, a proven technique for working with microbiome data.

Deep neural networks are particularly useful for working with microbiome data because of their ability to draw out meaningful patterns and create abstract, low-dimensional representations known as deep embeddings. The networks enable learning tasks on large data inputs such as microbiome profiles reporting trillions of microbe abundances present.

We discuss a multi-input neural network model that characterizes microbiome profiles from fecal samples of deployed service members and integrates data from completed profile of mood states (POMS) questionnaires and blood panel tests. The model encompasses a pre-trained vision transformer (ViT) that uses Red Green Blue images representing microbial abundances and a multi-layer perceptron (MLP) that uses the POMS and blood results. The ViT is initially trained with 12,190 public human microbiome samples, and the overall model is trained to predict POMS responses and characterize samples based on deployment periods.

This work highlights the use of a novel neural-driven approach that employs multimodal data sets and provides an efficient way of characterizing health states in deployed environments. Our model shows the promise of leveraging large sets of public data and novel data modes for prediction and characterization tasks using methods that may not be possible via isolated data representations.

THE ARTGUT *IN VITRO* SYSTEM

Todd Thorsen , Catherine Cabrera , Isabel Smokelin , James Comolli , Johanna Bobrow , Elif Kilic , Katherine Besse and David Walsh (MIT Lincoln Laboratory)

Human gut microbiome studies today are almost exclusively limited to animal models due to a lack of available in vitro tools capable of recreating the complex gut environment. Furthermore, the over-simplification of existing in vitro tools reduces the utility and predictive value of the data they generate. Building upon the growing MITLL expertise in 3D printing, we have been designing and prototyping milliliter-scale ArtGut systems with 90 independent chambers that emulate the gas gradients found in the colon that enable the co-culture of microbiome communities with diverse oxygen requirements. These initial prototyped parts, 3D-printed in rigid photosensitive urethane-based materials, function as bioreactors in our ongoing study to investigate the impact of environmental perturbations on key metabolic profiles of polymicrobial cultures. Dysbiosis triggered by a change in environmental conditions (such as exposure to chemical toxins) can have nuanced, but potentially serious effects on the host. Current microbiome studies on toxicants rely on methods such as 16S sequencing and short-chain fatty acid (SCFA) analysis. Using ArtGut as the primary culture tool, we are taking this analysis to the next level to identify key metabolic pathways impacted by dysbiosis. Using back-end tools like flow injection analysis electrospray ionization mass spectrometry (FIA ESI MSMS) to analyze the culture samples, we are able to detect microbiome metabolites at single micromolar concentrations, orders of magnitude lower than the millimolar concentrations typically observed in the colon. Combining this data with metatranscriptomic genetic analysis of the microbiome samples pre- and post-exposure to chemical toxins provides a detailed snapshot of key pathways activated, and allows the benchtop researcher to investigate potential mitigation treatments in subsequent ArtGut experiments.

Technical Session #4: Remediation

THINKING GLOBALLY ABOUT MARINE MICROBIAL COMMUNITIES ON FUTURE U.S. NAVY COATINGS

Melissa R. Kardish, U.S. Naval Research Laboratory

To increase speed, maneuverability, and performance of its ships while reducing costs, the U.S. Navy must reduce or eliminate biofouling from seawater-exposed ship hull surfaces. New coating formulations are developed to improve performance and to combat changing conditions and biofouling communities. The next generation of coating approaches attempt to reduce fouling through foul-release and/or anti-fouling formulations. Larger macrofouling on ship hulls that causes substantial drag and can damage hulls is proceeded by microbial biofilms. To understand the early development of these communities, identify precursors to more problematic macrofouling, and compare responses in different climates and seasons, we sampled biofilms on emerging coating technologies at global locations. In coordination with the Intersite Calibration effort led by NSWC Carderock, we have begun a coordinated sampling effort where we sample 12 coatings (including 9 test coatings) across 6 ONR field test sites spanning the Atlantic and Pacific Oceans. I will present the insights we learned from the first few of months of sampling off these panels. I will discuss the similarities and differences we see in global patterns across 12 surface types and describe our future sampling plans. Results from these early studies may be able to give insights about performance and mechanisms of biofouling.

BIOSTIMULATION OF RDX DEGRADATION IN TWO RANGELAND SOILS

- Chris Baker (US Army CRREL)
- Flora Laurent (US Army CRREL)
- Alison Thurston (US Army CRREL)
- Stacey Doherty (US Army CRREL)
- Robyn Barbato (US Army CRREL)

RDX (Royal Demolition eXplosive) is a common soil and groundwater contaminant at US military installations, where it poses known or potential threats to the environment and human health. Natural breakdown of RDX by microbes near the soil surface is limited, despite several RDX-metabolizing enzyme systems having been identified in bacteria and fungi at contaminated sites. Furthermore, efforts to remediate contaminated soil by augmenting or stimulating soil microbes have met with mixed success. In this study, we set out to explore ways to increase soil surface RDX breakdown. We used experimental microcosms to examine the separate and interactive effects of (i) different soils, (ii) contamination with RDX, (iii) inoculation with the RDX-degrading bacterial strain Rhodococcus rhodochrous 11Y, and (iv) augmentation with compost, on soil microbial communities and RDX levels. Following experimental manipulations, we used high-performance liquid chromatography (HPLC) to quantify RDX concentrations over time;quantitative PCR (qPCR) to quantify abundance of bacteria and of the xplA gene involved in aerobic RDX breakdown;and amplicon sequencing to characterize the makeup of the bacterial community. Adding R. rhodochrous 11Y sped up RDX degradation compared to uninoculated microcosms, with the contaminant mostly removed from inoculated microcosms within five weeks, compared to seven weeks for uninoculated microcosms. Adding compost had mixed effects on RDX degradation rates. Interestingly, in contrast to most microcosms, samples from one location without added compost or R. rhodochrous 11Y showed almost no RDX breakdown and very low levels of the xplA gene in microcosms. This result suggests that natural soil microbiomes may sometimes lack RDX-degrading bacteria, but this deficiency may be overcome by inoculation with a specific strain or community with RDX-degradation capacity.

EXPLORING THE CORROSION MICROBIOME OF COPPER-NICKEL ALLOYS IN NATURAL SEAWATER

Rachel L. Mugge (National Research Council Post-Doc, Naval Research Laboratory, Ocean Sciences Division), Treva T. Brown (Naval Research Laboratory, Ocean Sciences Division), Jason S. Lee (Naval Research Laboratory, Ocean Sciences Division)

Copper-nickel (CuNi) alloys are used extensively throughout the Navy and marine industry in shipboard seawater piping systems and heat exchangers because these alloys provide excellent corrosion and macrofouling resistance, and thermal conductivity. However, microbiologically influenced corrosion (MIC), a combination of microbiological, chemical, and electrochemical processes, can lead to accelerated deterioration and severe damage of CuNi materials. While MIC has been extensively studied, knowledge gaps still exist which connect specific microorganisms and changes in seawater chemistry to corrosion initiation and propagation. Examining the corrosion microbiome of newly formed biofilms on CuNi alloys under relevant operational conditions in natural seawater is necessary to improve knowledge on this mechanism of deterioration. To this end, a series of field experiments were designed to mimic shipboard conditions of seawater piping systems. Four alloys, including three CuNi alloys (UNS C70600, UNS C71600, UNS C72200) and titanium (CP grade 2) as a control, were exposed to natural seawater and sediment collected from Port Everglades, FL. Operational conditions including sediment content, flow rate, dissolved oxygen concentration, and

exposure time were varied during each experiment. At the conclusion of each short-term exposure, corrosion biofilms were aseptically sampled, DNA was extracted, and 16S rRNA gene sequencing was performed to discover differences in bacterial community composition, abundance, and diversity. The initial and post-experiment seawaters from the bioreactors were collected and chemically and biologically characterized. Additionally, corrosion product chemistry was quantified using energy dispersive spectroscopy, and corrosion morphology was characterized using scanning electron microscopy. Data from these experiments, particularly the corrosion microbiome, will improve knowledge of predictive capabilities and potential preventative measures within CuNi systems susceptible to MIC attack.

CHARACTERIZING POLYMER VALORIZATION BY MICROBIAL FUNGI ISOLATED FROM AIRCRAFT USING TIME-SERIES NASCENT RNA-SEQ ANALYSIS

Dominique N. Wagner, Victor Roman, Blake Stamps

Polymer degradation by microbes is often considered a problematic phenomenon in the built environment, but this same capability could be engineered for waste valorization of polymers. Fungi, along with other microbes, can degrade various materials, include many polymers. By characterizing this process, we can engineer biological polymer waste valorization in a controlled and targeted manner. This will allow for more effective polymer recycling efforts and the regeneration of base feed stocks for new polymer materials. This capability is especially relevant for remote terrestrial infrastructure and space operations. Here, we describe a method for characterizing the gene expression cascade that leads to the degradation of a polymer coating. By using emerging nascent RNA to capture gene expression as it is occurring, we can use a novel causal inference machine learning algorithm to map the gene expression cascade of microbes in response to environmental stimuli. This method relies on an initial dimensionality reduction followed by the causal inference analysis to eliminate autocorrelation and capture true cause-effect signals within microbes and microbial communities. By mapping a complete gene expression cascade we can target gene or processes that are vital to polymer degradation and could be made more effective through biological engineering.

Technical Session #5: Health and Performance 2

ALCOHOL INFLUENCES GUT MICROBIOME: RESULTS AND IMPLICATIONS FOR UNITED STATES MILITARY MEMBERS AND VETERANS

Andrew J. Hoisington(1-3), Christopher A. Stamper(1,2), Kelly A. Stearns-Yoder (2) , Elizabeth J. Kovacs (4), Lisa A. Brenner1-2,5,6
(1)Department of Physical Medicine & Rehabilitation, University of Colorado Anschutz Medical Campus, Aurora, CO, USA
(2)VA Rocky Mountain Mental Illness Research Education and Clinical Center (MIRECC), Rocky Mountain Regional VA Medical Center (RMRVAMC), Aurora, CO, USA
(3)Department of Systems Engineering & Management, Air Force Institute of Technology, Wright-Patterson AFB, OH, USA
(4)Department of Surgery, University of Colorado Anschutz Medical Campus, Aurora, CO, USA
(5)Department of Psychiatry, University of Colorado Anschutz Medical Campus, Aurora, CO, USA
(6)Center for Neuroscience, University of Colorado Anschutz Medical Campus, Aurora, CO,USA

Alcohol use in the military, while once viewed as an integral aspect of service, is now known to result in negative physical and psychological health outcomes. The United States (US) Center for Disease Control and Protection survey noted that the military is the heaviest drinking profession. Research, including that conducted by this research team, have consistently identified alcohol use as a driver of microbial diversity. Herein we summarize the results of three studies of alcohol and the gut microbiome. In our United States Veteran Microbiome Project (US-VMP), we observed a lower abundance of Akkermansia for current alcohol use disorder (AUD) participants, as compared to those with no history of AUD. Moreover, those with current AUD had gut microbiomes more associated with psychological disorders (e.g., PTSD, generalized anxiety disorder, major depressive disorder). In contrast, participants with no history of AUD had gut microbiomes more associated with demographic (e.g., gender, ethnicity, race) and life events (e.g., homelessness, student status, education level, employment). Our study on mild traumatic brain injury showed participant with AUD, as compared to those without, had significantly lower alpha-diversity and the beta-diversity for weighted UniFrac approached significance. In another of our trauma related studies, we grouped participants into high and low alcohol levels and observed in that post-burn trauma study, the two groups had microbial community differences that were close to significance, partially driven by the low alcohol group with elevated abundance of Akkermansia and Bacteroides. Repeatably finding Akkermansia in our studies is notable because it is an intestinal commensal which promotes barrier function in part by enhancing mucus production. Overall, our findings highlight an urgent need to further investigate alcohol use, its influence on the gut microbiome, and identify systematic targets for improved health for our military and Veterans.

SHIGELLOSIS RESULTS IN PERSISTENT CHANGES TO THE GUT MICROBIOME

Zachary Liechty[1,2], Ariana Baldwin[1], Sandra Isidean[3], Eddie Suvarnapunya[4], Robert Frenck[5], Michael Goodson[1], Chad Porter[3]
[1]Human Performance Wing, Air Force Research Lab
[2]UES, a Blue Halo Company
[3]Naval Medical Research Command
[4]Walter Reed Army Institute of Research
[5]Cincinnati Children's Hospital

Shigella is a significant cause of dysentery predominantly affecting children in middle- and low-income countries, as well as international travelers. While the primary symptoms caused by Shigella or other diarrhea-inducing pathogens resolve within a few days, persistent molecular and physiological changes can occur in infected individuals. Infection can lead to a reduced gut permeability, increased likelihood of developing irritable bowel syndrome, and increased prevalence of antimicrobial resistance genes. While some reports have demonstrated pathogen-induced diarrhea can alter the gut microbiome, no studies have examined the effects of Shigella inoculation on microbiome composition in a controlled infection setting. Here, we profiled the fecal microbiomes of 45 individuals infected with Shigella sonnei 53G before infection, during infection, and after antibiotic treatment. This model allowed for a detailed exploration of microbiome temporal dynamics during infection, as well as a comparative analysis between those who did and did not experience severe symptoms (shigellosis). We found that alpha diversity decreased to a greater degree in individuals with shigellosis and that decreases in alpha diversity significantly correlated with the relative abundance of Shigella. Furthermore, perturbations in microbial composition during infection compared to the pre-infection state were significantly larger in individuals diagnosed with shigellosis than those who were not. After recovery (28 days after infection), those with shigellosis still had persistent changes to their microbiomes while those without shigellosis recovered to a composition

resembling their pre-infection microbiome. Shigellosis-induced changes include an increased abundance of multiple ASVs classified as Lachnospiraceae and Veillonella, and decreased abundance in ASVs classified as Alistipes, Faecalibacterium, and Oscillospiraceae. Correlation analysis also identified various ASVs that increased in abundance alongside Shigella, including Strepotcoccus ssp., Veillonella ssp., and Hemophilus parainfluenzae. This study elucidated the dynamics of the gut microbiome under Shigella infection, and identifies an altered state of the microbiome after recovery that could lead to insights about long-term issues arising from Shigella infection and potential interventions to lessen its burden.

MICROBIOME DYNAMICS IN SWINE WOUND MODEL: EVALUATING TOPICAL NONEUPHORIC PHYTOCANNABINOID ELIXIR 14 AGAINST STANDARD SILVER DRESSING

Aarti Gautam[1], Joshua Stephenson[1], Allison Hoke[1], Nabarun Chakraborty[1], Kristo Nuutila[2], Anders Carlsson[2,3], and Rasha Hammamieh[1]

[1]. Medical Readiness Systems Biology, CMPN, Walter Reed Army Institute of Research, Silver Spring, MD.

[2]. United States Army Institute of Surgical Research, 36950 Chambers Pass, Fort Sam Houston, TX 78234, USA.

[3]. Metis Foundation, 84 NE Interstate 410 Loop Suite 325, San Antonio, TX 78216, USA.

Introduction: Thermal injuries are caused by exposure to a wide variety of agents including heat, electricity, radiation, chemicals, and friction. Early intervention can decrease injury severity by preventing excess inflammation and mitigating thermal injury progression for improved healing outcomes. The standard point of care Silverlon®, when activated by moisture, generates silver ions that may be effective against a wide spectrum of wound pathogens and can influence composition and dynamics of the wound microbiome. Cannabinoids possess anti-inflammatory properties and there is no information available related to its role in wound microbiome. Therefore, the purpose of this study is to investigate wound microbiome post topical application of Silverlon® and Noneuphoric Phytocannabinoid Elixir 14 (NEPE14) containing a full complement of phytocannabinoids (< 0.3% delta-9-tetrahydrocannabinol or cannabidiol) and other phytochemicals.
Methods: Deep partial-thickness thermal wounds were created on the dorsum of four anesthetized pigs and treated with NEPE14, Vehicle control, Silverlon®, or gauze within one hour post injury. The biopsies were collected post on days 0, 4, and 14. Microbiome analysis (16s DNA) was conducted on these tissues using standard Illumina procedure using Miseq instrument (Illumina). The data was processed using standard in-house pipeline after mapping the sequences on Greengenes database.
Results: Significant differences were observed between the Silverlon® and NEPE-14 treatments at all timepoints assessed. The standard care of treatment SilverIon® exhibited the lower diversity compared to the NEPE-14 treatment. The phyla Proteobacteria and Firmicutes had the highest prevalence, followed by Actinobacteria and Bacteroidetes. In terms of wound healing and presence of bacteria as assessed using the MolecuLight imaging system (MolecuLight Inc,Toronto, ON, Canada), no statistically significant differences were observed.
Conclusions: The antibacterial properties of Silverlon® naturally resulted in reduced biodiversity when compared to NEPE-14. Interestingly, despite lacking known antibacterial properties, NEPE-14 inhibited the growth of Firmicutes at different timepoints and warrants further investigation.
Disclaimer: Material has been reviewed by the Walter Reed Army Institute of Research. There is no objection to its presentation and/or publication. The opinions or assertions contained herein are the private views of the author, and are not to be construed as official, or as reflecting true views of the Department of the Army or the Department of Defense. Research was conducted under an IACUC-approved animal use protocol in an AAALAC International - accredited facility with a Public Health Services Animal Welfare Assurance and in compliance with the Animal Welfare Act and other federal statutes and regulations relating to laboratory animals.

IMPACT OF MELANIZED BACTERIA ON THE INTESTINAL HOMEOSTASIS AND MICROBIOME: POTENTIAL PROBIOTIC FOR TREATMENT OF RADIATION INJURY AND INFLAMMATION DISEASES

Zheng Wang

Radiation damage and oxidative stresses in the intestinal mucosa are linked to inflammation and immunity, impacting gut microbiota. It is needed to develop medical countermeasures targeting the microbiome to mitigate or treat radiation injuries and inflammation diseases. Melanin has been demonstrated a unique capacity to scavenge reactive oxygen species (ROS) in vitro and in vivo. We hypothesize that targeting gut microbiome through melanized bacteria can mitigate and treat host from oxidative and radiation damages. We engineered the commensal bacterium E. coli Nisssle strain that synthesizes melanin and assessed its safety in targeting the gut microbiome to prevent such damages. We administered both melanin and melanized bacteria to mice and monitored its impact on intestinal physiology and body weight. We noted an increase in the abundance and colonization of melanized E. coli Nissle in the intestines, while the compositions of certain gut microbiome components remained unchanged. No significant changes in body weight or intestinal inflammation were detected in the mice treated with the melanized bacteria. These findings confirm the safety of melanin-producing bacteria as probiotics, supporting further research into treating radiation-induced injuries and gut inflammation diseases.

Poster Session Abstracts

Poster No.	Title	Presenting Author
TSMC 8-01	The new power couple: integrating machine learning with soil microbial fuel cells for near real-time detection of subsurface contaminants	Katie L. Duggan DiDominic
TSMC 8-02	Changing temperature and moisture conditions of an Arctic Finnish Soil alters both microbial activity and community composition	Lindsay Gaimaro
TSMC 8-03	Comparative Analysis of Leading Wastewater Monitoring Approaches to Interrogate Gastroenteritis at U.S. Air Force Academy	Cullen M. Ingersoll
TSMC 8-04	The Isolation and Characterization of Active and Dormant Microorganisms in Ice-Rich Permafrost	Joy O'Brien
TSMC 8-05	Agnostic Pathogen Sensing Using Wastewater Biosurveillance	Katherine Besse
TSMC 8-06	Seasonal Taxonomic and Diversity-based Analysis of Chesapeake Bay Microbiomes	Charles R. Sweet
TSMC 8-07	Exploring MICP at Low Temperatures: Harnessing Cold-Adapted Microorganisms	Alison K. Thurston
TSMC 8-08	Impact of Copper in Early Biofilm Community Development	Sara M. Tuck
TSMC 8-09	Hunting the wild unicorn: Efforts to clone uncharacterized endotoxin-modifying bioactivities	Timothy D. Wright
TSMC 8-10	The Role of Biosafety in Microbiome Reseach	Akwasi Agyeman
TSMC 8-11	Characterization of the gut microbiota in the Aotus nancymaae non-human primate diarrhea model for improved evaluation of antidiarrheal therapeutics	Carlos Gaspar
TSMC 8-12	To what extent does V. fischeri use T6SS weapons to invade an already colonized territory?	Aundre Jackson
TSMC 8-13	Field expedient stool collection methods for gut microbiome analysis in deployed military environments	Car Reen Kok
TSMC 8-14	Microbially Induced Calcium Carbonate Precipitation Compressive Strength is Affected by Substrate Particle Size in Biocement Bricks	Hannah Grover
TSMC 8-15	Withdrawn	-
TSMC 8-16	A multi-strain human skin microbiome model provides a testbed for disease modeling	Angela Maloney
TSMC 8-17	Metagenomic Databases, Curation, and Data Fusion Tools for Predicting Military-Relevant Human Health States	Jose Manuel Martí

Poster No.	Title	Presenting Author
TSMC 8-18	Discovery metabolomics to identify key difference in the fecal metabolomes of soldiers as biomarkers of predictive performance assessments	Whitman, Jordan A
TSMC 8-19	The effects of prolonged isolation on animal and human host microbiota diversity	Morie Alpha
TSMC 8-20	Characterizing the effects of bacteriophage treatment for Pseudomonas aeruginosa infection on the microbiome of the model organism Caenorhabditis elegans	CDT Similoluwa Olaniyi
TSMC 8-21	The Role of Domestic and Wild Canine Species in Zoonotic Disease Dynamics	Aaron Kim
TSMC 8-22	Withdrawn	-
TSMC 8-23	Analysis of bronchoalveolar lavage fluid metatranscriptomes among patients with COVID-19 disease	Michael Jochum
TSMC 8-24	Impact of spaceflight-induced stress on the gut-brain axis in a mouse model.	George Dimitrov
TSMC 8-25	Impact of radiation dose and TPOm medical countermeasure on fecal microbiome in a mouse model	Allison Hoke
TSMC 8-26	Quantifying Short Chain Fatty Acid Production Potential in the Gut Microbiome	Anika Havlik

TSMC 8-01: THE NEW POWER COUPLE: INTEGRATING MACHINE LEARNING WITH SOIL MICROBIAL FUEL CELLS FOR NEAR REAL-TIME DETECTION OF SUBSURFACE CONTAMINANTS

Katie L. Duggan DiDominic, Robert M. Jones, Robyn A. Barbato, Randall W. Reynolds, Scott M. Slone
US Army Engineer Research and Development Center (ERDC) Cold Regions Research and Engineering Lab (CRREL)

To understand the efficacy of soil microbial fuel cells (SMFCs) to function as biosensors for contaminants and of machine learning algorithms (MLAs) to decipher environmental perturbations (gross contamination) in near real time, we designed and implemented a series of flow-through SMFCs to simulate the conditions of field deployment and to investigate the influence of subsurface drainage on the microbial activity driving their performance. A total of 8 SMFC microcosms were constructed and given a 10-day colonization period. Afterward, 4/8 SMFCS will be the control group and be treated with only deionized water at discreet intervals. Simultaneously, a separate 4/8 SMFCs will be treated with alternating deionized water and urea solution to represent uncontaminated and contaminated wetting events. The voltage of each SMFC will be continuously monitored autonomously and the MLA will be tasked with interpreting the voltage patterns to identify when a contamination driven shift in voltage has occurred. While this study is on-going, preliminary data suggests that SMFCs will respond dynamically to wetting events, with urea contaminated substrate causing new voltage trends following wetting events in comparison with the controls (i.e., DI water). We suspect that additional contamination events will cause the urea SMFCs to stray further from the pre-contamination voltage trends, while responses seen in controls may return to a near pre-wetting

condition. These expected differences in response will improve the capability of the MLA to decipher between a rain event (i.e., control) and the introduction of a contaminant by comparing the new voltage output to that of an earlier time point.

TSMC 8-02: CHANGING TEMPERATURE AND MOISTURE CONDITIONS OF AN ARCTIC FINNISH SOIL ALTERS BOTH MICROBIAL ACTIVITY AND COMMUNITY COMPOSITION

Lindsay Gaimaro, Stacey Doherty*, Chris Baker*, Robyn A. Barbato**
**ERDC Cold Regions Research and Engineering Laboratory, Hanover, NH*

More frequent periods of warmth and precipitation in the Arctic are leading to increased fluctuations in soil temperature and moisture conditions. Slight changes in soil abiotic conditions can alter microbial activity, as observed through respiration measurements and extracellular enzyme activity (EEA) analyses that determine enzyme potential. To understand the effects of soil temperature and moisture on CO2 flux and EEA, we prepared soil collected from the Finnish Arctic for a laboratory incubation study by wetting it to different moisture contents and incubated at various temperatures. We hypothesized that increased temperatures would linearly correlate with both increases in microbial respiration and EEA due to corresponding increases in metabolic activity. Conversely, we expected to see a Gaussian relationship between soil moisture content and microbial respiration and EEA. Lastly, we expected to see shifts in the soil microbial communities from changes in both soil temperature and moisture conditions. Microbial respiration and EEA significantly correlated with higher soil temperatures, while microbial respiration negatively correlated with moisture content at warmer temperatures. Only the nitrogen degrading EEA potential activity was significantly impacted by both temperature and moisture conditions, with activity peaking around field moist conditions (10 kPa). As expected, microbial beta diversity shifted with temperature and clustered separately by moisture content at warmer temperatures. The microbial alpha diversity, however, decreased with temperature. These data support hypotheses that microbial communities will become more active in a warmer and wetter Arctic and that nitrogen cycling will potentially increase with changing soil conditions.

TSMC 8-03: COMPARATIVE ANALYSIS OF LEADING WASTEWATER MONITORING APPROACHES TO INTERROGATE GASTROENTERITIS AT U.S. AIR FORCE ACADEMY

Cullen M. Ingersoll[1]; Riley E. McGarry[1]; John P. Collins[2]; Xiang-Jun Lu[2]; Alper Gokden[2]; Anastasia Cuff[2]; Bryant J. Webber[3]; Andrew B. Wallace[3]; J. Jordan Steel[1]; J. Kenneth Wickiser[2,4]; Armand L. Balboni[1], Michael A. Mechikoff[1]
[1] Department of Biology, Life Sciences Research Center, United States Air Force Academy, 2355 Faculty Dr Ste 2N375, USAF Academy, CO, 80840 USA
[2] Global Alliance for Preventing Pandemics, Center for Infection and Immunity, Mailman School of Public Health, Columbia University, 722 W 168th St. New York, New York, 10032 USA
[3] 10th Operational Medical Readiness Squadron, United States Air Force Academy, 4102 Pinion Dr, USAF Academy, CO 80840 USA
[4] Heilbrunn Department of Population and Family Health, Mailman School of Public Health, Columbia University, New York, New York

Wastewater monitoring offers the unique ability to surveil pathogen prevalence in a population at a given time and identify health risks that may affect mission readiness. Currently, pathogen monitoring is primarily done with qPCR which provides quantitative data but is limited by the number of pathogens targeted. Alternatively, panel-based systems like BioFire cannot support quantified data

and only provide detection of categorically grouped species (GI, upper respiratory, etc.), making it more suited for clinical use investigating pathogens linked to known symptoms. Whole Genome Sequencing (WGS) allows for metagenomic analysis of a whole sample, but requires difficult bioinformatic analysis, particularly in wastewater, when excess genetic data from sources such as human or animal floods the data of interest. Unconstrained by the shortcomings of WGS, BioFire, or qPCR, Virome Capture Sequencing (VirCapSeq-VERT) and Bacterial Capture Sequencing (BacCapSeq) use Next Generation Sequencing and biotinylated probe sets to capture coding regions of genomes of all vertebrate viruses, core regions of pathogenic bacteria, and known AMR markers and isolate them from most excess genetic material for sequencing. Here, we use and compare qPCR, BioFire and VirCapSeq to monitor USAFA wastewater for gastroenteritis-causing agents. Trends of adenovirus, astrovirus, and norovirus were common and with VirCapSeq's ability to capture all vertebrate viruses, further analysis of pathogens such as mammalian orthoreovirus are being investigated. Future clinical assessment may reveal if the presence of these viruses is indicative of infected humans or animals at USAFA.

TSMC 8-04: THE ISOLATION AND CHARACTERIZATION OF ACTIVE AND DORMANT MICROORGANISMS IN ICE-RICH PERMAFROST

Joy O'Brien, Indiana University, Bloomington, IN USA
Robyn Barbato, Cold Regions Research and Engineering Laboratory, Hanover, NH USA

Permafrost—permanently frozen soil in Arctic and Antarctic regions—is thawing due to increasing temperatures caused by climate change. Thawing permafrost, especially ice-rich permafrost, presents unique challenges to infrastructure, terrain, and ecosystem structure and function all of which are vital to the success of military operations. One of the concerns accompanying thaw is the permafrost microbiome, a diverse microbial community composed of psychrophilic bacteria equipped with robust survival strategies. Previous studies have demonstrated that the permafrost microbiome composition changes due to thaw yielding differences in contribution to biogeochemical cycling which can cause an increase in microbial respiration, further accelerating thaw. Here, we focus on the isolation and characterization of psychrophilic bacteria from intact ice wedge (15,000-20,000 YBP) and permafrost samples (30,000-40,000 YBP) from the CRREL Permafrost Tunnel in Fairbanks, AK. Specifically, we aimed to collectively isolate 10 members from phyla Acidobacteriota and Bacillota at 4°C and 14°C, as these bacteria have repeatedly been found in previous studies to be abundant in permafrost affected soils before and after a thaw event. We targeted the isolation of Acidobacteriota via minimal medium and soil extract medium and Bacillota by heat-treating prior to plating for isolation, which kills vegetative cells leaving bacterial endospores intact. These isolates and others from the permafrost microbiome can provide insight into biotechnological applications, as many are capable of forming endospores, specialized proteins to aid in cold stress, and the production of pigments which can mitigate extensive UV exposure and act as an antimicrobial agent.

TSMC 8-05: AGNOSTIC PATHOGEN SENSING USING WASTEWATER BIOSURVEILLANCE

Katherine Besse , Joseph Lacirignola , Trina Vian , Amy Xiao , Johanna Bobrow , Tony Mannion , James Stylinski , Carolyn Alphen , Henry Lau , Charles Sinkler , Jason Jong , Kajal Claypool , Diane Jamrog , David Walsh , Emily Kurdzo (MIT Lincoln Laboratory)

Wastewater-based epidemiology (WBE) has emerged as an insightful field of study for microbial and

pathogen tracking since its use during the COVID-19 pandemic. Since then, the field has expanded from viral surveillance to include a host of other pathogens and targets, including antibiotic resistance markers and community immune response. Despite these advancements, the field as a whole is still disconnected and lacks consistent protocols for these investigations, which vary widely and are often manual and time-intensive. In addition, there is a lack of understanding around current wastewater monitoring technology capabilities as the field moves towards agnostic pathogen sensing rather than species-targeted methods, such as qPCR. Our DOD-funded program aims to tackle these two major issues by developing a field-forward prototype that utilizes metagenomic sequencing to get a broad look into the bugs that exist in wastewater. In its first year, the program performed a systems analysis of wastewater architectures and built a virtual sewer system model to assess and inform the technology requirements for the prototype we plan to build. The systems analysis model was validated by laboratory-based experiments and provided proof of concept for the overall project vision. Here we will present these findings and speak to the program's next steps.

TSMC 8-06: SEASONAL TAXONOMIC AND DIVERSITY-BASED ANALYSIS OF CHESAPEAKE BAY MICROBIOMES

Charles R. Sweet[1†], Ahren W. Jin[1,2], Courtney Chandler[3,4], Caitlyn J. Koo[1,2], Logan M. Treaster[1,5], and Robert K. Ernst[3]

† to whom correspondence should be addressed: sweet@usna.edu
[1] – United States Naval Academy (USNA) Chemistry Department, Annapolis MD
[2] – present address: Uniformed Services University of the Health Sciences (USUHS), Bethesda MD
[3] – University of Maryland School of Dentistry Department of Microbial Pathogenesis, Baltimore MD
[4] – present address: National Institutes of Health, Bethesda MD
[5] – present address: University of Kansas Medical School, Wichita KS

The Chesapeake Bay is the largest estuary in the United States and is of ecological, economic, and strategic importance. We have conducted a multi-year effort to determine the structure of the planktonic bacterial microbiome in both the Middle Bay (Annapolis region) and the Severn River by bioinformatic analysis including taxonomic and diversity-based assessment. These datasets demonstrate characteristic differences by season in the composition of the environmental microbiome using several approaches, including culture-based characterization and metagenomic full-shotgun analysis of the whole community. The winter microbiome is more uniform in taxonomic diversity than the summer one both between and within years, a commonality that suggests the winter microbiome is repopulated yearly from a persistent biological reservoir;preliminary data and further work address the hypothesized origin of this reservoir.
In this project we compare 16S culture-based microbiome characterization with full-shotgun NGS metagenomic methods including read-based, contig-based, and genome-based classification schemes. The culturable cohort analysis is based on a 1000+ strain collection we have curated from this watershed in both winter and summer over several years. This collection reveals that our understanding of the culturable environmental microbiome is not yet saturated, as approximately one third of these organisms are novel species. This strain collection will serve as a resource for exploration of novel biocapabilities present in the environmental microbiome in future work.

TSMC 8-07: EXPLORING MICP AT LOW TEMPERATURES: HARNESSING COLD-ADAPTED MICROORGANISMS

Alison K. Thurston ERDC – CRREL; Seung Oh ERDC – CERL; Hyunjung Kim ERDC- CERL; Logan Gonzalez ERDC - CRREL

David Walker ERDC – EL; Scott Michael Slone ERDC – CRREL; Dale Landas ERDC – CERL; Robyn Barbato ERDC – CRREL; Donald Cropek ERDC - CERL

Microbial induced carbonate precipitation (MICP) is a ubiquitous bio-geochemical process, resulting in the precipitation of calcium carbonate. MICP has garnered attention as a potentially sustainable low-carbon emission alternative to conventional cement. Current research predominantly focuses on a few mesophilic bacteria and temperatures exceeding 10°C in engineering applications overlooking the biological aspects driving this process. These gaps hinder the scalability of MICP technology, impede control over crystal growth and strength, and limit its application in extreme temperature environments. To address these limitations, we have isolated approximately 50 environmental organisms and evaluated their potential to perform MICP at cold temperatures (<10°C). Currently, we have narrowed down our selection to eleven candidates. Our research goals are to:
1. Perform a phenotypic analysis of the cold environment candidate microorganisms and identify genes responsible for MICP.
2. Characterize urease activity and $CaCO_3$ crystal formation under varying environmental conditions (e.g., varying temperature).
3. Identify candidate mutations for protein engineering to improve MICP at cold temperatures.
Through this multidisciplinary approach, we aim to advance our understanding of MICP, paving the way for its broader adoption in sustainable construction and environmental remediation.

TSMC 8-08: IMPACT OF COPPER IN EARLY BIOFILM COMMUNITY DEVELOPMENT

Sara M. Tuck[1,2], Melissa R. Kardish[1], Gary J. Vora[1], Katherine J. Franz[2], Kenan P. Fears[1]

[1] Center for Biomolecular Science and Engineer, Naval Research Laboratory, Washington, DC
[2] Department of Chemistry, Duke University, Durham, NC

Biofouling, the accumulation of unwanted organisms on submerged assets, is an ongoing challenge within the maritime industry and has additional repercussions on human health. Biofouling build-up increases fuel consumption, asset drag, and operational costs in addition to facilitating the transfer of environmental and pathogenic bacteria from one location to another. Conventional methods to inhibit biofouling includes the application of antifouling coatings, the most popular of which are copper based. In biological systems, copper is tightly regulated and, in an attempt to exploit this, some antifouling coatings contain up to 75% copper (I) oxide by weight. Despite these high loadings, the efficacy of these coatings is rapidly declining with the emergence and spread of copper tolerant species. Microbial communities resistant to copper have been found to form mature biofilms on these coatings, which could be altering the interfacial properties to create more favorable conditions for the settlement of a broader biofouling community. To gain an understanding of the mechanisms responsible for the loss of antifouling performance, coated and uncoated polyvinyl chloride panels were deployed at field sites to harvest early biofilms. From these collections, we isolated, cultured, and identified bacterial species. Copper tolerance profiles were developed by re-exposing individual colonies to copper sulfate in broth microdilution assays. We also investigated copper biocide release from copper-ablative coated glass coverslips over a short time frame to better understand the copper environment that is susceptible to primary colonization.

TSMC 8-09: HUNTING THE WILD UNICORN: EFFORTS TO CLONE UNCHARACTERIZED ENDOTOXIN-MODIFYING BIOACTIVITIES

Timothy D. Wright, United States Naval Academy Chemistry Department
Ian O'Keefe, University of Maryland School of Dentistry Department of Microbial Pathogenesis
Hyojik Yang, University of Maryland School of Dentistry Department of Microbial Pathogenesis
Robert K. Ernst, University of Maryland School of Dentistry Department of Microbial Pathogenesis
Charles R. Sweet, United States Naval Academy Chemistry Department

Lipid A is a fundamental component and membrane anchor of Gram-negative endotoxin (lipopolysaccharide, LPS). This molecule also comprises the essential outer leaflet of the Gram-negativeouter membrane. It is, therefore, of structural importance to the bacterial cell and is a primary surface forenvironmental and host-pathogen interactions. The basic biosynthetic scheme for synthesizing thismolecule (Raetz biosynthetic pathway) has been known for over twenty years, however work continues inexploring the extensive variety of both constitutive and regulated modifications of this molecule acrossthe diversity of phylogenetic clades and evolutionary niches in which it occurs. Francisella andPseudomonas are two important genera in which the bioactivities responsible for endotoxin assembly andremodeling are still not fully elucidated. This is of particularly critical interest in Francisella, of whichthe species tularensis is a Tier I CDC Bioterrorism Agent.To explore these capabilities we will use rapid spectral characterization method we have designatedFast Lipid Analysis Technique (FLAT), applied to novel members of these genera recently isolated fromour Chesapeake Bay environmental microbiome and known to make uncharacterized structuralmodifications of interest. We will take a variety of approaches (including directed cloning informed bywhole genome-sequencing and functional investigation of a cosmid library) to identify unknown LPS-modifying enzymes including possible phosphatase, glycosylase, and/or hydroxylase targets. Success inthis effort will resolve long-standing biosynthetic questions in the field and may contribute new insightsto the endotoxin Structure-Activity Relationship.

TSMC 8-10: THE ROLE OF BIOSAFETY IN MICROBIOME RESEARCH

Akwasi Agyeman, Ph.D., MPH, and Dennis Pak.
711 Human Performance Wing/Institutional Review,
Air Force Research Laboratory
Wright-Patterson Air Force Base, Ohio.

The 711 Human Performance Wing (HPW) Biosafety Office advises the 711 HPW investigators, collaborators, and AFRL Technical Directorates that have a memorandum of agreement with the 711 HPW on matters involving laboratory biological safety. It coordinates the transfer of biosafety information to personnel and standing committees that overlap with biosafety issues and makes biohazard policy consistent with DoD, Federal, State, and Local laws, regulations, and guidelines. It provides expedient and proactive consultation and review for investigators, laboratory staff, and the research community at large. Different biosafety regulations, guidelines, and steps taken to provide guidance to mitigate risks will be discussed.

TSMC 8-11: CHARACTERIZATION OF THE GUT MICROBIOTA IN THE AOTUS NANCYMAAE NON-HUMAN PRIMATE DIARRHEA MODEL FOR IMPROVED EVALUATION OF ANTIDIARRHEAL THERAPEUTICS

Carlos Gaspar - Vysnova Partners LLC. Alexandria, VA
Nereyda Espinoza - U.S. Naval Medical Research Unit SOUTH, Lima, Peru
Hugo Valdivia - U.S. Naval Medical Research Unit SOUTH, Lima, Peru

Rosa Nunez - U.S. Naval Medical Research Unit SOUTH, Lima, Peru
Mark Klempner - Department of Medicine, University of Massachusetts Chan Medical School, Worcester, MA
Lisa A. Cavacini - Department of Medicine, University of Massachusetts Chan Medical School, Worcester, MA
Tyler D. Moeller - Walter Reed National Military Medical Center, Bethesda, MD, USA.

The U.S. Naval Medical Research Unit SOUTH (NAMRU SOUTH) has developed a non-human primate (NHP) diarrhea model in the Aotus nancymaae that mimics human diarrheal disease caused by the main bacterial enteropathogens: Enterotoxigenic Escherichia coli (ETEC), Shigella and Campylobacter. This NHP diarrhea model has been shown to be a robust, reproducible and reliable model for the evaluation of new bacterial therapeutic countermeasures. Recently, non-antibiotic therapies have been associated with changes in gut microbiome composition. The gut microbiota acts as a physical barrier preventing colonization by pathogens and produces antimicrobial compounds. The symbiotic relationship between the immune system and gut microbiota shapes a healthy gut microbial ecosystem. Currently, non-antibiotic antidiarrheal therapies are being developed including passive immunity prophylactics and biotic-supplement oral solutions supplemented that could alter the microbiota. NAMRU SOUTH has evaluated the composition and diversity of the microbiome in Aotus to develop a gut modulation model to investigate the interplay between new therapies and the microbiome. Collected fecal samples were analyzed using 16s RNA sequencing. The dominant phyla were Firmicutes, Proteobacteria, Actinobacteria, Bacteroidetes and Fusobacteria. Among these, Bacteroidetes and Firmicutes predominated. In collaboration with the University of Massachusetts, we evaluated the effect of treatment with an anti-ETEC secretory immunoglobulin A on the intestinal microbiota. The immunoglobulin was shown not to perturb the gut microbial population underscoring its potential as a safe and effective targeted therapeutic approach in challenged animals. These first results highlight that Aotus can be a model to evaluate the effects of antidiarrheal therapies on the gut microbiota.

TSMC 8-12: TO WHAT EXTENT DOES V. FISCHERI USE T6SS WEAPONS TO INVADE AN ALREADY COLONIZED TERRITORY?

Aundre Jackson/UNC Chapel Hill, Alecia Septer/UNC Chapel Hill

Competition among co-occurring bacteria can change the composition and role of a microbial community. We use the connection between bioluminescent bacteria and their squid host to study how environmentally transmitted bacteria contend for a limited number of host colonization sites. Vibrio fischeri use a type VI secretion system (T6SS) to eliminate competing strains from co-habiting sites in the host. This allows for bacteria to engage in fatal battles as they attempt to establish a beneficial association. We modified our culture-based competition assays to mimic a scenario where a competitor that is susceptible to T6SS mediated killing (ES114) dominates the territory and the invader (ES401) must use its T6SS weapon to take over. We co-incubated differentially tagged competitor strains at a 100:1 ratio to give the target strain (ES114-GFP) the advantage over the invader strain (ES401-RFP). The invader strain had either a functional T6SS weapon, or a mutation that disarmed the strain. Coincubations between the target and armed or disarmed invaders were performed in 6 well agar plates that were diluted each day with liquid media to mimic the daily venting of the host that dilutes the symbiotic population each day. These dilutions were then placed onto fresh agar plates to mimic regrowth. The process was repeated daily over a 94-hour period. We tracked each strain in the coincubation spot by imaging for green and red fluorescence over time in coculture to see if the killer strain would take over. Within a 94-hour period ES401 had completely taken over and eliminated the target ES114, while the disarmed mutant did not increase in numbers.

Taken together, these results indicate that a T6SS armed strain can invade and take over already occupied territory in culture. Current work is exploring invasion dynamics with the natural host of V. fischeri, E.berryi squid.

TSMC 8-13: FIELD EXPEDIENT STOOL COLLECTION METHODS FOR GUT MICROBIOME ANALYSIS IN DEPLOYED MILITARY ENVIRONMENTS

Car Reen Kok, Lawrence Livermore National Laboratory, Livermore, CA
James B. Thisse, Lawrence Livermore National Laboratory, Livermore, CA
Michele Cerroni, Infectious Disease Clinical Research Program, Department of Preventive Medicine and Biostatistics, Uniformed Services University of the Health Sciences, Bethesda, MD
David R. Tribble, Infectious Disease Clinical Research Program, Department of Preventive Medicine and Biostatistics, Uniformed Services University of the Health Sciences, Bethesda, MD
Tahaniyat Lalani, Infectious Disease Clinical Research Program, Department of Preventive Medicine and Biostatistics, Uniformed Services University of the Health Sciences, Bethesda, MD
Nicholas A. Be, Lawrence Livermore National Laboratory, Livermore, CA

Advancement of microbiome research in military deployed environments requires proper collection and storage of samples. This is particularly challenging in remote deployed environments whereby cold chain storage and transport is not possible. Prior research has discussed the impact of fecal collection methods on microbiome composition and stability and have assessed the potential use of Flinders Technology Associates (FTA) cards and OMNIgene (OG) gut kits as deployable methods in the field. In our study, we utilized fecal samples collected during an ongoing clinical trial evaluating gut health during military deployment and travel to regions with risk of traveler's diarrhea. We performed shotgun metagenomic sequencing and compared microbiome composition of paired fecal samples collected using FTA cards (n=49) and OG kits (n=49) across timepoints (pre- and during-travel) and across subjects that were either asymptomatic or experienced travelers' diarrhea. Higher concentrations of nucleic acid and sequencing library were observed in OG samples. Furthermore, differences in microbial diversity and composition between FTA and OG were apparent whereby an enrichment of Actinobacteria species was observed in FTA samples. In addition, we observed biases of each collection method towards specific species whereby skin microbes such as Kytococcus were uniquely captured by FTA while environmental species such as Nautilia were captured by OG. These observations implied the need for standardized protocols within field studies and the potential influence of fecal collection methods on across study comparisons. Overall, the data presented here will help the design of fecal microbiome study protocols in field and military deployment settings.

TSMC 8-14: MICROBIALLY INDUCED CALCIUM CARBONATE PRECIPITATION COMPRESSIVE STRENGTH IS AFFECTED BY SUBSTRATE PARTICLE SIZE IN BIOCEMENT BRICKS

Hannah Grover - United States Air Force Academy, Nikolas Schwendeman - United States Air Force Academy, Melanie Grogger - United States Air Force Academy, Life Science Research Center, Victoria Morrison - United States Air Force Academy, J. Jordan Steel - United States Air Force Academy

Biocementation uses the biological functions and processes of enzymes, bacteria, and cellular structures to mineralize and bind particles and produce aggregate materials. Sporosarcina pasteurii (S. pasteruii) is a ureolytic microbe that can precipitate $CaCO_3$, calcium carbonate, through a process called microbially induced calcite precipitation (MICP). The ureolytic bacteria breaks down urea into

carbonate and ammonium. Calcium addition will enable calcium carbonate to link surrounding particles together. The purpose of this study is to determine if substrate particle size affects Biocementation. Based on preliminary work, we hypothesize that a larger starting substrate particle size will produce the strongest aggregate material. The results from this study will be advantageous for future Air and Space Force Operations such as dust stabilization in spacecraft landings and substrate aeration in Lunar and Martian agricultural operations. The Biocementation process has already been used in military operations;however, the most abundant form of Martian regolith is around 50 to 150 µm in diameter. Such sand is much finer than that on Earth which ranges from 0.02 to 2 mm in diameter. For this reason, the question of whether Biocementation is feasible and robust enough to support Space Operations must be explored.

TSMC 8-15: WITHDRAWN

TSMC 8-16: A MULTI-STRAIN HUMAN SKIN MICROBIOME MODEL PROVIDES A TESTBED FOR DISEASE MODELING

Angela Maloney, ; Tyler Crawford, ; Jordan Hurlbut, ; Monica Martinez, ; TJ Mulhern, ; Vidhya Vijayakumar, ; Elizabeth Wiellette, ; Else Vedula, ; Draper, Cambridge, MA

The skin microbiota serves critical roles at the interface between the human body and the environment, providing resistance to pathogenic strains, building host immunity, and supporting epithelial turnover. In addition, the skin microbiome has been characterized as dysbiotic in response to host disease states. One approach to characterize microbial and host biology is to derive in vitro models of sufficient complexity and stability to support perturbation and response. Current tools for studying these processes are focused on testing two or more strains for short culture durations, thereby precluding studies of relevant complexity and chronic disease states. Here, we present an in vitro model of the human skin microbiome comprising a 6-strain consortium colonizing primary human keratinocyte tissue for up to 7 days. Established readouts include histology, gene expression, and transepithelial electrical resistance (TEER), as well as relative strain abundance to characterize microbiome stability over time. Skin cells formed a complex tissue structure over two weeks and maintained stable or increasing TEER after 7 days of co-culture with the microbial consortium. Up to 5 strains were viable on the keratinocyte surface on day 7 as validated by custom qPCR assays demonstrating a robust and stable testbed for microbiome studies. A remarkable feature of this model is the persistence of Cutibacterium acnes in aerobic tissue culture environment, suggesting that the skin tissue model is conducive to more natural growth states of native skin strains. The addition of cytokines representative of atopic dermatitis (AD) elicited a marked alteration in relative strain abundance in diseased model tissues, demonstrating capability to study the impact of disease states on the microbiome and vice versa. We envision this model system as a test bed to evaluate the influence of commensals on host biology, the influence of external environment on microbiome stability, and chronic diseases impacted by dysbiosis.

TSMC 8-17: METAGENOMIC DATABASES, CURATION, AND DATA FUSION TOOLS FOR PREDICTING MILITARY-RELEVANT HUMAN HEALTH STATES

Jose Manuel Martí, Car Reen Kok, James Thissen, Nisha Mulakken, Aram Avila-Herrera, Jonathan E. Allen, Crystal Jaing, Nicholas A. Be

A multitude of disease states are associated with the microbiome, positioning it as a target with tremendous potential for proactively predicting and improving human health and resilience. Leveraging the breadth of metagenomic data for military and civilian health, however, has been hampered by the data processing, curation, and harmonization required for functionalizing microbiome information into predictive tools.

To facilitate comprehensive sequence assignment, we created an nt-based index for the Centrifuge metagenomic classifier. This database, which includes references spanning all kingdoms of life, was subjected to robust quality control filtering and reference decontamination, thereby minimizing spurious taxonomic classifications. We then curated and processed 13K+ publicly available raw metagenomes associated with a range of disease states, employing our nt database for metagenomic classification. Each resultant metagenome profile was assigned a curated “health” or “disease” label, in addition to a consistent structure of accompanying metadata. Finally, we created a web-based framework for access to the resultant taxonomic and metadata profiles. This robustly curated dataset represents a rich resource for training and testing predictive models for predicting military and deployment-relevant disease states, including gut health disruption, infection, and neurological impacts.

Our metagenomic classification pipelines and data fusion product provide a curated, structurally consistent package of microbiome features associated with health or disease states. We intend this resource to spur future advances in microbiome machine learning, with the aim of constructing models tuned for predicting military-relevant health states, improving health and readiness for the warfighter.

TSMC 8-18: DISCOVERY METABOLOMICS TO IDENTIFY KEY DIFFERENCE IN THE FECAL METABOLOMES OF SOLDIERS AS BIOMARKERS OF PREDICTIVE PERFORMANCE ASSESSMENTS

Whitman, Jordan A.;Straight, Chad R.;Doherty, Laurel A, Pantoja-Feliciano de Goodfellow, Ida G.;Giles, Grace E, Soares, Jason W

Cognitive function is a critical determinant of Soldier performance in operational settings. Emerging evidence indicates that intestinal microbiota influence cognition via the gut-brain axis, meaning these metabolites could serve as key biomarkers of performance in Soldiers and lead to novel intervention targets. However, metabolomics of fecal samples only canvasses molecules that are unabsorbed by the host, providing no insight into the functional capacity of the gut microbiome. In vitro fermentation methods allow us to circumvent these limitations by modelling the functional capacity of the microbiome in real-time and discover potential gut-derived biomarkers associated with cognitive performance, including those absorbed by the host in vivo. In this study, fecal samples were collected from 24 male soldiers and batch fermentations were conducted to generate time-dependent fermentates. Untargeted metabolomics analysis using UPLC MS/MS was employed to study changes in the fecal metabolite profile from baseline (0 hr) to 24 hr. Soldiers were divided into tertiles (Above Average (AA);Average (A);Below Average (BA)) based on performance from a battery of cognitive tasks, including decision-making, memory, and spatial cognition under stress. Preliminary baseline findings revealed >100 unique metabolites in each performance groups, from metabolite classes like amino acids, hormones, ketones, nucleotides and fatty acids. Additional down selection is

ongoing to identify significant changes over the 24 hr fermentation in the three groups. This study represents an initial discovery of biomarkers that may be indicative of higher performers within cognitive tasks and serve as future targets for predictive performance assessments. Further research is needed to increase sample size, validate the associations of the discovered metabolites to cognitive outcomes, and refine the selection of metabolites.

TSMC 8-19: THE EFFECTS OF PROLONGED ISOLATION ON ANIMAL AND HUMAN HOST MICROBIOTA DIVERSITY

Morie Alpha and Ian Squires

We assessed the effects of prolonged isolation on animal and human host microbiota diversity, and its impact on health outcomes, by analyzing a series of studies based on animal and human microbiome data. Infectious disease studies of individual hosts examining the correlation between isolation and host microbiota diversity have generally shown that isolation is usually associated with dysbiosis and negative health outcomes. But there a sever lack of comprehensive meta-analysis of data from both individual and cohort studies of animals and humans to test the hypothesis that isolation could lead to microbiota diversity loss. To test this hypothesis, we derived estimates of microbiota diversity changes using data from 17 animal isolation studies, and four human isolation studies. The animal studies compared the microbial diversity of animals in the wild with animals in captivity (or domesticated animals). The human studies compared changes in microbiota during prolonged isolation. Participants in the human studies were held in varying degrees of isolation that ranged from 150 days to 520 days. Results from our estimates showed no clear evidence of microbial diversity loss in animals or humans during isolation from their respective natural environments. On the contrary, we observed a trend towards moderate increase in microbiota diversity of human subjects after prolonged isolation. We also saw a similar pattern with the animal studies;they either showed no changes or a slight increase in microbiota diversity in captive animal populations after prolonged isolation from their natural environments. The general trend towards an increase in microbial diversity during isolation is worth exploring further to determine the confounding factors undermining the expected microbiota decline. An understanding of the factors causing an increase in microbial diversity during isolation could result in the measurement and mapping of microbial flow, which could provide opportunities for the development of appropriate biodefense measures with minimal health consequences.

TSMC 8-20: CHARACTERIZING THE EFFECTS OF BACTERIOPHAGE TREATMENT FOR PSEUDOMONAS AERUGINOSA INFECTION ON THE MICROBIOME OF THE MODEL ORGANISM CAENORHABDITIS ELEGANS

CDT Similoluwa Olaniyi ('26)1, CDT Eric Song ('26)1, CDT Aidan Tran ('25)1, 2LT Katherine Hebert, CDT Shreyan Mitra ('27)1, Dr. Caroline Amoroso1, Dr. Chris Kovacs1, Dr. Jennifer Dumaine1, LTC Andrew Kick1
Department of Chemistry and Life Science, United States Military Academy (USMA), West Point, NY1

The prevalence of multidrug resistant bacteria (MDR) has increased in recent years, representing a global public health risk that makes infections harder to treat and other medical procedures much riskier. Within the military population, MDR bacteria pose a serious threat to service members with combat related injuries. Resistance to existing antibiotics is emerging faster than new antibiotics can be discovered, highlighting the need for additional therapeutic options for MDR pathogens.

Bacteriophages are viruses that infect bacteria. Inside the bacterial cell, lytic bacteriophages grow, replicate, and ultimately lyse the cell to spread the virus to a new host. As a result, there has been growing interest to use bacteriophage as a treatment option for MDR bacterial infections. While it is understood that bacteriophage can be used as a targeted approach to kill bacteria, there has been little investigation into the consequence of bacteriophage treatment on the microbiome of the individual receiving treatment. To explore the effects of bacteriophage treatment on the gut microbiome, we utilized a Caenorhabditis elegans infection model because the gut of the nematode is sterile until colonized by the bacteria it consumes as food and because native microbiome of the organism has been published. For our studies, C. elegans were first colonized with nonpathogenic bacteria that normally comprise the microbiome through culture on plates seeded with a bacterial cocktail lawn and allowing worms to feed. Following establishment of the microbiome, C. elegans were infected with Pseudomonas aeruginosa (PA14), a lethal bacterial pathogen of C. elegans, for 24 hours before treatment with PA14 lytic bacteriophage identified and isolated from wastewater samples in Cornwall, NY. Phage treated C. elegans demonstrated significantly higher survival rates than untreated worms, and bacteriophage treatment was specific for PA14 in the C. elegans gut. These preliminary results will be expanded as we develop more complex microbiome models with increased diversity in bacterial species populating the gut to recapitulate what is happening in human infections. Furthermore, we will characterize phage-microbiome interactions and explore the contribution of the host immune system to bacterial clearance. Finally, we will apply this system to other phage-pathogen combinations.

TSMC 8-21: THE ROLE OF DOMESTIC AND WILD CANINE SPECIES IN ZOONOTIC DISEASE DYNAMICS

Aaron Kim, India Jones, Kellie M. Kuhn
U.S. Air Force Academy

The domestication of animals has had fundamental and far-reaching implications for the evolution of infectious diseases in humans. By bringing animals together in higher densities and in close association with human communities, humans established a pathway for disease transmission. Among domestic animals, dogs are a major reservoir for zoonotic infections. Dogs are capable of transmitting zoonoses to humans through bites, scratches, or contact with saliva or feces. With an estimated 900 million dogs on the planet, it is not surprising that humans and dogs exchange a large number of pathogens. Inadequate vaccination and preventive care for dogs can exacerbate health risks. In this study, we are examining the microbiome of canids to detect potential threats to public health. To quantify canine-associated microbial communities, we will collect fecal samples from domestic dogs, feral dogs, and wild canids. We will use shotgun metagenomics to characterize the microbial communities associated with domestic and wild canines. We predict that the prevalence of pathogens and parasites will be determined by the animals' lifestyle, diet, and vaccination status. Examination of the microbial dynamics of human-canine relationships can help advance our understanding of the consequences of dog domestication on human and animal health and wellbeing.

TSMC 8-22: WITHDRAWN

TSMC 8-23: ANALYSIS OF BRONCHOALVEOLAR LAVAGE FLUID METATRANSCRIPTOMES AMONG PATIENTS WITH COVID-19 DISEASE

Michael Jochum;Division of Maternal-Fetal Medicine, Department of Obstetrics and Gynecology, Baylor College of Medicine, Baylor College of Medicine, Texas Children's Hospital, Houston, TX, 77030, USA
Michael D. Lee;Blue Marble Space Institute of Science, Seattle, WA, 98104, USA
Kristen Curry;Department of Computer Science, Rice University, Houston, TX, 77005, USA
Victoria Zaksas;Center for Translational Data Science, University of Chicago, Chicago, IL, 60615, USA
Clever Research Lab LLC, 2501 Chatham Rd, Suite N, Springfield, IL, 62704, USA
Elizabeth Vitalis;Inscripta, Inc, 5500 Central Ave STE 220, Boulder, CO, 80301, USA
Todd Treangen;Department of Computer Science, Rice University, Houston, TX, 77005, USA
Kjersti Aagaard;Division of Maternal-Fetal Medicine, Department of Obstetrics and Gynecology, Baylor College of Medicine, Texas Children's Hospital, Houston, TX, 77030, USA
Krista L. Ternus;Signature Science, LLC, 8329 North Mopac Expressway, Austin, TX, 78759, USA

To better understand the potential relationship between COVID-19 disease and hologenome microbial community dynamics and functional profiles, we conducted a multivariate taxonomic and functional microbiome comparison of publicly available human bronchoalveolar lavage fluid (BALF) metatranscriptome samples amongst COVID-19 (n = 32), community acquired pneumonia (CAP) (n = 25), and uninfected samples (n = 29). We then performed a stratified analysis based on mortality amongst the COVID-19 cohort with known outcomes of deceased (n = 10) versus survived (n = 15). Our overarching hypothesis was that there are detectable and functionally significant relationships between BALF microbiomes and the severity of COVID-19 disease. We observed 34 functionally discriminant gene ontology (GO) terms in COVID-19 disease compared to the CAP and uninfected cohorts, and 21 GO terms functionally discriminant to COVID-19 mortality ($q < 0.05$). A Dirichlet multinomial mixtures clustering analysis resulted in a best model fit using three distinct clusters that were significantly associated with COVID-19 disease and mortality. We additionally observed discriminant taxonomic differences associated with COVID-19, CAP, and uninfected BALF. Some positive correlations between COVID-19 mortality include an increase in the in Sphingomonas, Variovorax, and taxa belonging to the order Bacteroidales. Collectively, while this data does not speak to causality nor directionality of the association, it does demonstrate a significant relationship between the human microbiome, COVID-19 and CAP. The results from this study have rendered testable hypotheses that warrant further investigation to better understand the causality and directionality of host–microbiome–pathogen interactions.

TSMC 8-24: IMPACT OF SPACEFLIGHT-INDUCED STRESS ON THE GUT-BRAIN AXIS IN A MOUSE MODEL.

Stacy-Ann Miller[1], George Dimitrov[1,4], Burook Misganaw[1,4], Alexander Lawrence[1,3], Allison Hoke[1], Stephen Butler[1,4], Aarti Gautam[1], Melissa A. Kacena[2], Nabarun Chakraborty[1], Rasha Hammamieh[1]

[1]Medical Readiness Systems Biology, CMPN, Walter Reed Army Institute of Research, Silver Spring, MD
[2]Department of Orthopedic Surgery, Indiana University School of Medicine, Indianapolis, IN
[3]ORISE, Walter Reed Army Institute of Research, Silver Spring, MD, USA
[4]Culmen International, Alexandria, VA

Microbiomes play a critical role in tempering host immunity and energy metabolism. It has been well established that the relationship between the gut and the brain (gut brain axis) goes both ways. We hypothesize that the composition of gut microflora is susceptible to musculoskeletal surgery and its subsequent disuse. Consequent changes in bioenergetics play major role in healing, and the underlying molecular mechanisms associated with the gut microbiome and the brain needs to be further examined.

A segmental bone defect model (SBD) with ketamine/xylazine to anesthetize was utilized on male C57BL/6j mice aged 6-8 weeks participating in the NASA/Rodent Research 4 (RR4) program. Fecal samples were subjected to shotgun sequencing and LC-MS based global metabolomics. Brain tissues were subjected to microarrays.

The study involved LC-MS based global metabolomics and sequencing fecal samples, identifying microbial genes and pathways, and analyzing taxonomic and phylogenetic classifications. It also identified metabolites and pathways linked to surgery and unloading. Image analysis showed that FLT-Saline patients experienced impaired bone healing compared to G-Saline patients. The study also identified microbiota markers linked to surgery and unloading. Microarray analysis on brain tissue revealed more gene changes in saline patients than in sham patients. The study suggests impaired healing of space-borne diseases may be influenced by gut microflora.

TSMC 8-25: IMPACT OF RADIATION DOSE AND TPOM MEDICAL COUNTERMEASURE ON FECAL MICROBIOME IN A MOUSE MODEL

Allison Hoke[1], Gregory Holmes-Hampton[2], Vidya P. Kumar[2], Ida Nela Crespo-Rosales[1,3], Aarti Gautam[1], Rasha Hammamieh[1], Sanchita P. Ghosh[2], and Nabarun Chakraborty[1]

[1]Medical Readiness Systems Biology, Walter Reed Army Institute of Research, Fort Detrick, MD, 21702

[2]Armed Forces Radiobiology Research Institute, Uniformed Services University of the Health Sciences, Bethesda, MD

3Culmen International, Alexandria, VA

Threat of radiation exposure and possible countermeasures is a concern as exposure even at reduced doses can lead to chronic illness known as Acute Radiation Syndrome (ARS). A TPOm drug has been shown to be a countermeasure to treat ARS in mice, thus we are investigating the long-term effects of different radiation doses with and without TPOm on the gut microbiome in a murine model of total body irradiation (TBI). Mice were divided into 4 groups and received 7.5 Gy TBI and saline vehicle, 8 Gy TBI and TPOm, 8.5 Gy and TPOm, or 0 Gy (Control), with the TPOm dose (1.0 mg/kg) 24 h before pre-exposure. Six months post-exposure, fecal pellets were collected and 16S libraries were sequenced on the Illumina MiSeq system. Sequenced reads were processed using QIIME2 and Greengenes2 database, PICRUSt2 was used to infer pathway abundances, and DESeq2 package in R was used for differential analysis. When compared to 0 Gy, one-way ANOVA of relative abundance of phylum- level taxa showed significant shifts at the highest TBI dosage of 8.5 Gy with TPOm. Alpha and beta diversity exhibited distinctions between the radiation groups and the controls, and numerous pathways were altered in response to the TBI with and without TPOm with significant taxa and pathways common among the radiation groups. Our results indicated that the microbiome experienced alteration 6 months after TBI with and without TPOm when compared to the 0 Gy control. Validation studies are ongoing.

TSMC 8-26: WARFIGHTER MICROBIOME: SHORT-CHAIN FATTY ACID GENE AMPLIFICATION

Havlik, A.[1], Kim, J.[2], Liechty, Z.[3,4], Baldwin, A.[4], & Goodson, M.S.[4]

[1]United States Air Force Academy, [2]Wright Scholar, Air Force Research Laboratory, [3]UES, A Blue Halo Company, [4]Air Force Research Laboratory

Short Chain Fatty Acids (SCFAs) have been identified as important microbially-produced effectors of gastrointestinal health and performance. Metagenomic, transcriptomic, and metabolomic analyses are the gold standards for SCFA quantitation within the gut microbiome. However, these methods are

expensive to perform. The purpose of this research project is to develop a digital polymerase chain reaction (dPCR)-based method to quantify changes in the potential for short-chain fatty acid (SCFA) production in microbiome samples to facilitate its inclusion in future microbiome research studies. We worked to optimize three primer sets (Ack, Mmd, and But) that were designed to amplify genes in the metabolic pathways producing the short-chain fatty acids acetate, propionate, and butyrate, respectively. These three SCFAs have been linked to protective factors against chronic disease and are known to be beneficial to overall health. Due to the high variability of these genes among various microbial taxa, an intercalating dye system was chosen for dPCR quantitation over a more specific primer-probe system in order to better capture this variation. However, the intercalating dye method meant off-target amplification and fluorescence would also be quantified in the dPCR analysis, leading to false positives. Therefore, the annealing temperature and other elements of the PCR protocol were optimized to prevent nonspecific binding of the primers. We used a gradient PCR to test the annealing of the primers to the desired gene at a range of temperatures and we then used gel electrophoresis to visualize the results. Since the length of the genes are known, gel electrophoresis and comparison against a DNA ladder reveals if the target gene has been amplified or if the primers are binding to other regions of the DNA. Once optimized for a single band, we tested the primers using dPCR, using a dilution series to identify if this method could be used to quantify the specific genes in a sample.

Currently, the ‘But’ primer has been optimized for dPCR but the optimization of the other two primers, Ack and Mmd, is ongoing.

This information will be used by the lab to conduct further research on how to optimize gut health and human performance through quantifying the production of SCFAs by gut bacteria. There is still much to learn regarding mechanisms that produce SCFAs in the gut, and the factors that impact optimal microbiome health, such as stress, diet, and environmental conditions. Further research is needed to leverage SCFA properties to improve cognitive capabilities, stress responses, emotional health, and physical readiness of the warfighter.
